# Speed of phototransduction in the microvillus regulates the accuracy and bandwidth of the rhabdomeric photoreceptor

**DOI:** 10.1371/journal.pcbi.1008427

**Published:** 2020-11-16

**Authors:** Roman V. Frolov, Irina I. Ignatova

**Affiliations:** Nano and Molecular Systems Research Unit, University of Oulu, Oulu, Finland; George Mason University, UNITED STATES

## Abstract

Phototransduction reactions in the rhabdomeric photoreceptor are profoundly stochastic due to the small number of participating molecules and small reaction space. The resulting quantum bumps (QBs) vary in their timing (latency), amplitudes and durations, and these variabilities within each cell are not correlated. Using modeling and electrophysiological recordings, we investigated how the QB properties depend on the cascade speed and how they influence signal transfer. Parametric analysis in the model supported by experimental data revealed that faster cascades elicit larger and narrower QBs with faster onsets and smaller variabilities than slower cascades. Latency dispersion was stronger affected by modification of upstream than downstream activation parameters. The variability caused by downstream modifications closely matched the experimental variability. Frequency response modeling showed that corner frequency is a reciprocal function of the characteristic duration of the multiphoton response, which, in turn, is a non-linear function of QB duration and latency dispersion. All QB variabilities contributed noise but only latency dispersion slowed and spread multiphoton responses, lowering the corner frequency. Using the discovered QB correlations, we evaluated transduction noise for dissimilar species and two extreme adaptation states, and compared it to photon noise. The noise emitted by the cascade was non-additive and depended non-linearly on the interaction between the QB duration and the three QB variabilities. Increased QB duration strongly suppressed both noise and corner frequency. This trade-off might be acceptable for nocturnal but not diurnal species because corner frequency is the principal determinant of information capacity. To offset the increase in noise accompanying the QB narrowing during light adaptation and the response-expanding effect of latency dispersion, the cascade accelerates. This explains the widespread evolutionary tendency of diurnal fliers to have fast phototransduction, especially after light adaptation, which thus appears to be a common adaptation to contain stochasticity, improve SNR and expand the bandwidth.

## Author summary

Phototransduction cascades in the rhabdomeric photoreceptors of invertebrates and the ciliary photoreceptors of vertebrates, while similar in their first stages, differ in their outcomes and regulation mechanisms. One salient difference is that in the photoreceptors of many insect species the delay of small responses decreases drastically after light adaptation. Here we performed an in-depth analysis of phototransduction in the insect microvillus and discovered hitherto unknown dependencies between the parameters of the cascade output, the quantum bump, and the cascade speed, supported by experimental data. Faster cascades produced faster, larger and narrower quantum bumps with smaller variabilities than slower cascades. We show that the differences in the cascade speed, either between species occupying dissimilar visual ecological niches, or between adaptation states, have profound consequences for photoreceptor signaling. Firstly, narrow and more synchronous bumps produced by fast cascades better transfer higher frequencies contained in visual signals, increasing the photoreceptor bandwidth. Secondly, a decrease in the level of noise due to the cascade acceleration compensates for the loss of signaling accuracy, which occurs when the signal duration decreases. We propose that acceleration of phototransduction is a powerful mechanism in both evolutionary and situational optimization of photoreceptor signaling.

## Introduction

Photoreceptors recode information from a time series of environmental photons into a continuous amplitude-modulated (graded) voltage response and then into many streams of synaptic vesicles stimulating the second-order visual neurons [1]. Due to the presence of various sources of noise, information is degraded at each transformation stage. This work is concerned with the loss of information at the initial stage of light-to-voltage transformation in the *rhabdomeric* photoreceptor caused by (1) the inherently random photon absorption events that can be described by a Poisson point process, and (2) the stochastic variability in the properties of elementary electrical responses, the quantum bumps (QB). These two sources of noise, correspondingly the extrinsic *photon* and the intrinsic *phototransduction* (or transduction) noises, change with light intensity and photoreceptor adaptation state, and represent two main determinants of the overall noise in the graded voltage response. They limit information transfer by the photoreceptor and generally the reliability of motion detection in the visual system [2].

The rate of information transfer by a photoreceptor stimulated with an arbitrary time series of light intensities representing a “pixel” of the visual scene dynamically sampled by the rhabdomere can be estimated using the Shannon’s equation [3]:

$$IR=\int_{f_{0}}^{f}\log_{2}\left[\frac{S(f)}{N(f)}+1\right]df$$ (1)

where *S*(*f*) and *N*(*f*) are signal and noise as functions of frequency, *f*, and their ratio is signal-to-noise ratio (SNR). The upper boundary of the photoreceptor signaling bandwidth, as measured by the low-pass corner frequency, is restricted by the characteristic duration of the QB, which is the widest in the dark-adapted and the narrowest in the light-adapted photoreceptors [4,5]. SNR in bright light depends as a function $1/\sqrt{n}$ on the number of operational sampling units, the microvilli. While the number of microvilli in photoreceptors of compound eyes of visually guided insects can vary from tens of thousands as in *Drosophila melanogaster* to hundreds of thousands and even millions as in *Periplaneta americana* [4,6], the size of their operational pool is usually smaller, because a fraction of microvilli is inactivated by preceding stimulation [4].

The useful frequency range of the photoreceptor is often flexible, with some of the flexibility traceable to the phototransduction cascade. This is best illustrated by comparing impulse responses evoked in photoreceptors in different adaptation states. Because Fourier transform of a momentary “impulse” stimulus contains all frequencies, the impulse response of the receptor contains all frequencies the system can process, i.e. the frequency-response range, and thus provides characterization of its current state, assuming the system’s stationarity and linearity. Although photoreceptors are inherently non-linear electrical systems [7], they can be considered linear when disturbed with stimuli that evoke small responses [8–11]. In a seminal study, Howard and colleagues [10] compared in vivo voltage impulse responses of dark- and light-adapted photoreceptors of several insect species. The impulse responses of photoreceptors after light adaptation accelerated dramatically, with their times to peak and durations decreasing strongly, especially in fast-flying diurnal species. However, a multiphoton voltage impulse response is a superposition of multiple QBs shifted relative to each other according to their individual latencies and filtered by the cell membrane. Although both the QB duration and latency dispersion are determined by the phototransduction, only the former was thought to be malleable, probably due to acceleration of inactivation of light-activated channels by elevated cytosolic calcium [4,9]. Low-pass filtering is also usually relaxed during light adaptation, speeding up both the onset and termination of light response because light-adapted cells are depolarized and their membrane resistance is lower than at rest [12].

This and the consecutive comparative studies [12–15] provided insights into the evolution of insect vision, by linking the speed of phototransduction cascade through biophysical properties of the light-insensitive membrane to visual ecology. Thus, in faster diurnal fliers, phototransduction is faster, generates smaller and narrower QBs, and adapts to a greater extent than in slower crepuscular flies. Photoreceptor membranes in the former are also less resistive than in the latter, preventing excessive low-pass filtering of the relatively fast light-induced currents. While this paradigm of matched filtering [16] explains evolutionary optimization of the frequency response range, which is the main determinant of information rate (Eq 1), little is known about mechanisms of noise optimization.

As mentioned above, the noise contained in the photoreceptor voltage response to light mainly originates from two sources, the variability in the number of absorbed photons when repetitively stimulated with the same stimulus and the variability in QB properties. These two dominate over other sources of noise, which include spontaneous activation of rhodopsins and Gq proteins, stochastic channel openings, noise from feedback synapses and gap junctions with the neighboring photoreceptors etc. [9,17–20].

Photon noise is discrete and conforms to the Poisson statistics (also referred to as photon ‘shot noise’ or ‘Poisson noise’). The variability it introduces is the largest at low photon counts, i.e. in dim light, and decreases as a function $1/\sqrt{n}$ of the number of photons in the stimulus. Depending on the duration and intensity of the stimulus, it can manifest in two extreme forms. If the stimulus is effectively an instantaneous “flash”, the photon noise will produce impulse responses comprised of variable numbers of QBs. If the stimulus is continuous and very dim, it will elicit a series of discrete QBs. The Poisson distribution will then describe the probability of encountering an impulse response consisting of *k* QBs in the first case and the probability for the occurrence of *k* responses per time interval in the second case.

Variabilities in amplitude, kinetics and timing of QBs that constitute the transduction noise [21–24] arise from the intrinsic stochasticity of phototransduction reactions and ion channel gating in the microvillus, due to the small numbers of molecules involved and small reaction space, the microvillus membrane area. Differences in biophysical properties or the functional states of the microvillus, e.g. variability in the expression of transduction proteins between microvilli or a degree of recovery from the previous excitation [4], may also contribute to variability. These variabilities degrade information transferred in impulse responses because the amplitudes and shapes of impulse responses elicited by the same stimulus and comprised of *the same number of QBs* will vary from trial to trial depending on the individual QB properties [17,18,24,25].

The quantitative relations between the transduction and photon noises in the rhabdomeric photoreceptor were first investigated by Laughlin and Lillywhite [17,18] in the seminal studies of dark-adapted locust photoreceptors stimulated by short stimuli with variable photon counts. It was shown that 1) the latency and amplitude dispersions are major components of the transduction noise, and 2) as the photon noise decreases with increasing stimulus intensity, the transduction noise becomes dominant. In a recent modeling study, Parag and Vinnicombe, by using an approach based on Bayesian point process (Snyder) filters, found that mean phototransduction delay (and not the QB variabilities) is the most important determinant of noise [26]. The modeling study by Abshire and Andreou suggested, under the assumption of noise additivity, that intrinsic noise sources tend to dominate over the photon noise in the bright light [27].

However, it is not known how different components of the transduction noise affect signaling in phototransduction cascades of different invertebrate species, how their contributions change with stimulation intensity and with changes in the adaptation state. Our previous study of the role of QB latency and its dispersion in signaling in the dark-adapted *Periplaneta americana* photoreceptors found that as mean photoreceptor QB latency (a proxy for the cascade speed) decreased, so did the latency dispersion [28], contrary to the established opinion that latency distribution does not change with mean latency [4,9]. This finding pointed to an additional mechanism, which could contribute to changes in both bandwidth and SNR during light adaptation.

The goal of the present work was to investigate as comprehensively as possible how properties of phototransduction and changes thereof affect signal processing. We therefore explored: 1) how the properties of QBs are connected to the properties of the phototransduction cascade, 2) how transduction noise arises from the stochasticity of the cascade, 3) how the QB properties and transduction noise influence signal transfer both in silico and in vivo, in different species and adaptation states, and 4) how transduction noise relates to the photon noise under different conditions. We found hitherto unknown correlations between QB parameters and that phototransduction shapes photoreceptor bandwidth and SNR via complex non-linear interactions between the characteristic QB duration and three QB variabilities. Our results suggest that light adaptation-dependent acceleration of phototransduction observed in many diurnal insect species represents a general adaptation to contain stochasticity and thus increase the speed and accuracy of photoreceptor signaling.

## Results

The Results section is structured as follows. In part 1, we investigate the dependencies of individual QB properties on the key events during the cascade. In part 2, we perform a parametric analysis of the cascade by studying the effects of systematic changes in five main activation parameters on the statistical properties of QBs. In part 3, we apply the results of the parametric analyses to the experimental data. In part 4, we explore how QB properties influence the corner frequency of signal transfer. In part 5, we exploit the discovered correlations between QB properties to estimate the transduction noise associated with responses of photoreceptors in vivo, under different adaptation conditions, and to compare it to the photon noise. Finally, in part 6, we investigate interactions between the sources of transduction noise and their effects on information capacity.

### Stochasticity in the phototransduction cascade

Three factors can contribute to the variability of QBs recorded from the same photoreceptor: the intrinsic stochasticity of the cascade, differences between microvilli, and differences in the functional states of microvilli in the rhabdom. The contributions of the first two factors cannot be easily separated because dispersions of QB amplitudes, durations and latencies arising from simulations of single microvillus responses match very well the dispersions found in recordings from individual photoreceptors [29]. Furthermore, recordings of QB trains originating from presumably the same microvilli in *D*. *melanogaster cam* mutant characterized by disrupted microvillus inactivation yielded amplitude and latency dispersions indistinguishable from those in wild-type flies [30]. The third factor depends on the degree of recovery of the microvillus from previous activation and can be considered negligible when dark-adapted photoreceptors are stimulated at frequencies of <2 Hz using flashes of light that evoke QBs with <50% probability.

**Fig 1:**
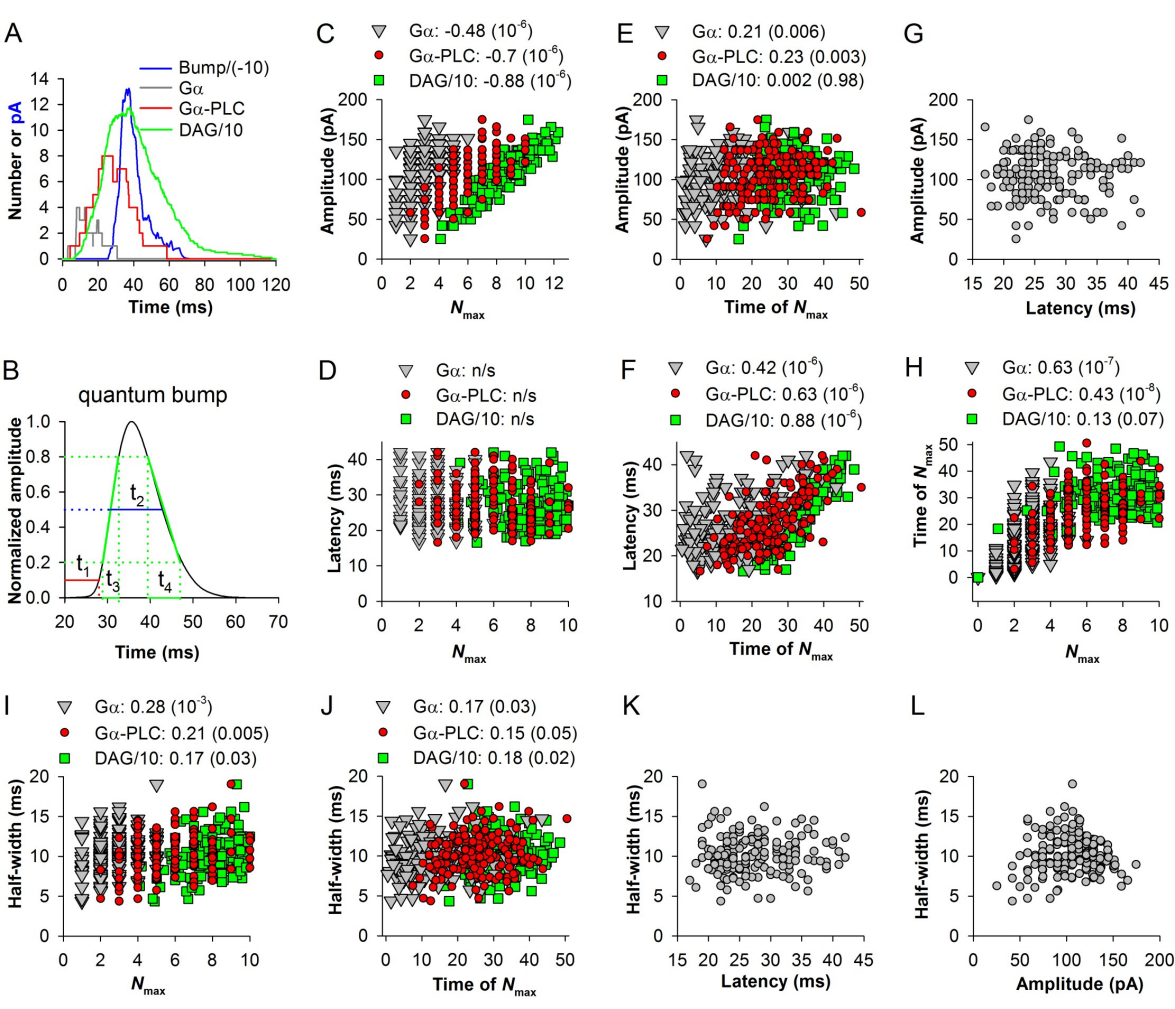
Speed, amplification and stochasticity in the “normal” *P*. *americana* cascade. The parameters of the model were adjusted to elicit current bumps closely resembling on average the in vivo *P*. *americana* current bump from the previous study [28]. Here and in all following simulations, the model was run 200 times, eliciting from ~130 to 198 bumps (depending on the parameter modification) that exceeded an amplitude threshold (here -7 pA). (A) Results of a single run of the model illustrate time-dependencies of Gα, Gα-PLC, DAG molecules and QB current; the DAG number and QB current were divided by 10 and -10, respectively, for presentation purposes. (B) Schematics of determining the following QB parameters: t_1_ (red) for 10% latency, t_2_ (blue) for half-width, t_3_ (green) for 20–80% rise and t_4_ (green) for 80–20% decay times. (C) Dependence of bump amplitudes on the maximal numbers (*N*_max_) of activated Gq proteins (Gα), or Gα-PLC complexes, or DAG molecules. Here, and in all following figures, unless stated otherwise, the first number in the title denotes the SROCC and the *P*-value is in the parentheses. (D) Bump latencies did not correlate with the maximum numbers of the precursor molecules; n/s stands for “non-significant” correlation. (E) Similarly, bump amplitudes either correlated weakly (for Gα-PLC) or did not correlate with the times of *N*_max_. (F) Correlations between the times of *N*_max_ and bump latencies. (G) Correlation between bump latencies and amplitudes. (H) Correlations between the *N*_max_ and times of *N*_max_ of Gα, Gα-PLC and DAG. (I, J) Bump half-widths correlated weakly or insignificantly with *N*_max_ (I) and the times of *N*_max_ (J) of Gα, Gα-PLC and DAG. (K, L) QB half-widths did not correlate with QB latencies (K) and amplitudes (L).

We therefore hypothesized that analysis of stochasticity using a mathematical model of QB could provide testable insights into the causes of QB parameter dispersions and possible correlations between them. We used a model that was originally developed by Nikolic et al. ([31]) for *D*. *melanogaster* and modified in the previous study to reproduce *P*. *americana* QBs [29]. All parameters used to produce the “normal” (henceforth “control”) cockroach current bump that deviate from the parameters of the original fly model are listed in S1 Table. In each experiment the computations were iterated 200 times, eliciting about 190 bumps. The results of one run showing dynamics of the activated Gq protein (Gα), activated phospholipase C (Gα-PLC), accumulation of diacylglycerol (DAG) and QB current are presented in Fig 1A. Due to stochasticity, the time-courses and amplitudes of the parameters varied strongly from run to run. We investigated if the times to peak and the maximal values (*N*_max_) of Gα, Gα-PLC and DAG correlate with the latency, amplitude and duration of the resulting QBs. Because the maxima of Gα and Gα-PLC can persist for some time and even be double-peaked, we used the times of the first observed maxima.

Fig 1B demonstrates the methodology of determining the QB parameters used in this study, including the 10% latency (t_1_), half-width (t_2_), and 20–80% rise (t_3_) and decay (t_4_) times. Fig 1C and 1D show dependencies of QB amplitudes and latencies during each run on *N*_max_ for Gα, Gα-PLC and DAG and Fig 1E and 1F on the corresponding times of *N*_max_. QB amplitudes were correlated with the *N*_max_ values (Fig 1C) but not with the times of *N*_max_ (Fig 1E). (In most simulations, the maximum number of activated channels was <50% of the maximal number of channels available, twenty five, preventing saturation-related artifacts.) In contrast, QB latencies were correlated with the times of *N*_max_ (Fig 1F) but not with *N*_max_ (Fig 1D). Interestingly, the strength of correlations in Fig 1C and 1F increased with the proximity of the cascade stage to the QB, indicating that the intrinsic randomness of molecular encounters and interactions tends to uncouple signal transfer and disrupt amplification.

One could expect that stronger amplification characterized by higher *N*_max_ values might be associated with longer times to *N*_max_, manifesting in a positive correlation between QB latency and amplitude. However, no such correlations could be detected in our simulated (Fig 1G) or experimental data. Plots of times to *N*_max_ against the respective *N*_max_ values for the three molecules (Fig 1H) explained the lack of such correlations as the strengths of correlations decreased consistently from the strong correlation for Gα to the statistically insignificant correlation for DAG. DAG is the immediate precursor of channel opening in the cascade, and so the absence of a correlation between *N*_max_ for DAG and the respective times to *N*_max_ predates the absence of the correlation between QB latency and amplitude. The third QB parameter, duration, can be approximated by the QB half-width. We explored correlations between the QB half-widths and other parameters (Fig 1I–1L). However, no significant correlations could be found.

### Parametric analysis of the cascade model

Next, we investigated how changes in five main activation parameters would alter timing, amplitude and duration of the QBs *on average*. The parameters were: (1) the time of guanosine diphosphate (GDP) to guanosine-5′-triphosphate (GTP) exchange by Gα, which we varied in 1 ms steps from 1 to 8 ms per event and presented in terms of rate in the remainder of the text; (2) the availability of Gq protein varied in steps from 300 to 10%; (3) the concentration of PLC varied similarly to Gq; (4) phosphatidylinositol 4,5-bisphosphate (PIP_2_) breakdown time by Gα-PLC mainly varied in 0.2 ms intervals from 0.1 to 2 ms per event and was also converted to rate; and (5) the sensitivity of light-activated channels to DAG, which we varied from 0.15 to 0.8. In this analysis we did not investigate the effects of changes in diffusion rates of Gαβγ, Gα and PIP_2_, because we found that such changes produce only minor effects [29].

**Fig 2:**
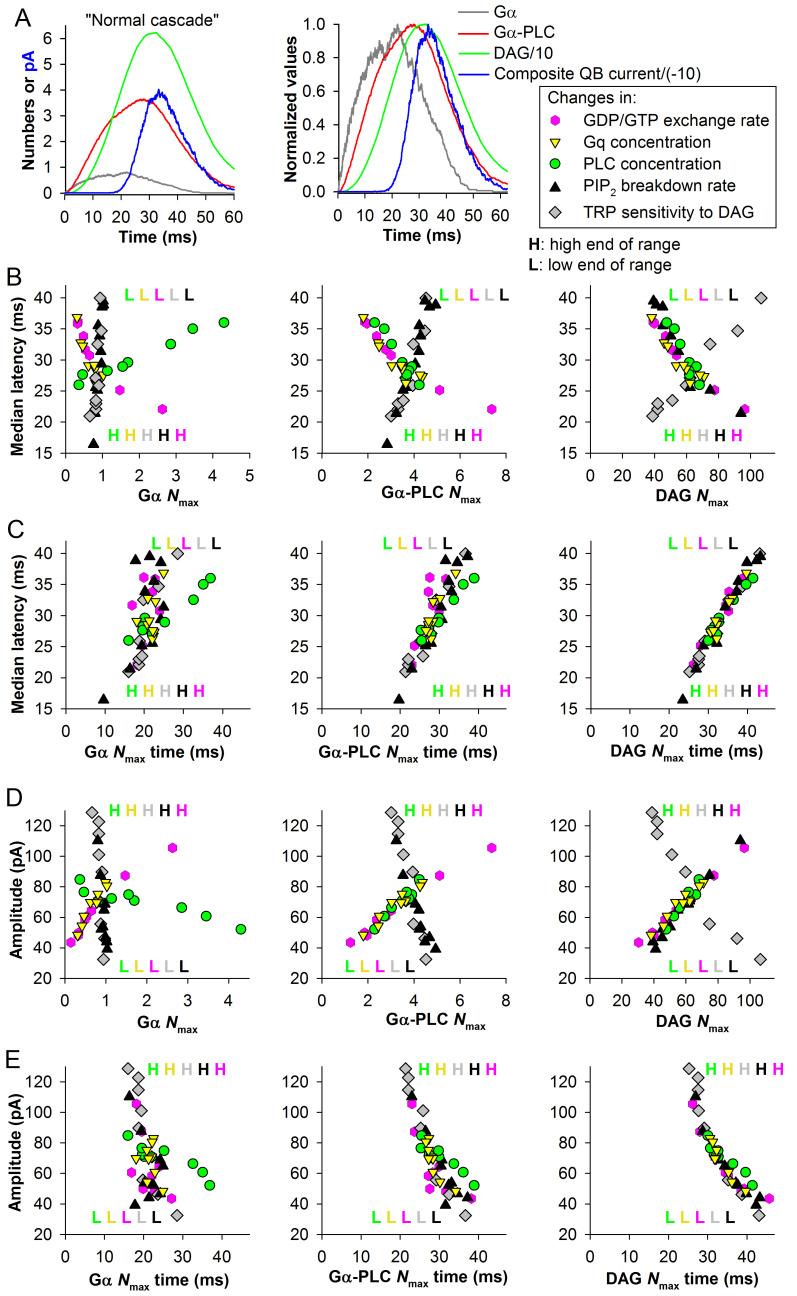
Parametric analysis of the cascade. (A) Left: average time-dependencies of Gα, Gα-PLC, DAG and quantum bump currents in the “normal” *P*. *americana* cascade from Fig 1; DAG and bump currents were divided by 10 and -10, respectively, as in Fig 1; data obtained in each run were averaged without alignment, hence the “composite QB current” term is used for consistency with the composite responses studied in next sections. Right: the normalized averaged time-dependencies. (B) Dependencies of median latency on the numbers (*N*_max_) of Gα, Gα-PLC or DAG molecules at the peak of the corresponding average curves as in A, upon incremental changes in five activation parameters. Here and elsewhere, GDP to GTP exchange rate was altered in the range from 125 to 1000 s^-1^ (control at 333 s^-1^); Gq and PLC concentrations from 10 to 300% (control at 100%); PIP_2_ breakdown rate by Gα-PLC from 500 to 10000 s^-1^ (control at 1428 s^-1^); sensitivity of light-activated channels to DAG from 0.15 to 0.8 (control at 0.34); here and in all following figures, color-coded “H” and “L” letters denote correspondingly the higher and lower limits of the parameter ranges in terms of either concentrations for Gq and PLC, or rates for GDP to GTP exchange and PIP_2_ breakdown, or sensitivity of the channels. (C) Dependencies of median latencies on the times of *N*_max_ of Gα, Gα-PLC or DAG curves. (D) Dependencies of mean bump amplitudes on the *N*_max_ of Gα, Gα-PLC or DAG curves. (E) Dependencies of mean amplitudes on the times of *N*_max_ of Gα, Gα-PLC or DAG.

The left panel in Fig 2A shows evolutions in time of *average* numbers of Gα, Gα-PLC and DAG molecules and also the QB current during the cascade under control conditions. The data were obtained by averaging all runs of the model that resulted in bumps, in this case 190 (a typical run is shown in Fig 1A) without alignment. The right panel of Fig 2A shows normalized curves to visualize the relative positions of the peaks.

Median latencies, mean amplitudes and mean half-widths were obtained from the simulated QB sets. Fig 2B and 2D demonstrates complex dependencies of median QB latency and mean amplitude, respectively, on the maximal numbers of Gα, Gα-PLC and DAG obtained from the peaks of the corresponding average time-dependencies. In general, increases in Gq and PLC concentrations, GDP to GTP exchange and PIP_2_ breakdown rates, and channel sensitivity to DAG were associated with faster, higher and more narrow bumps, and *vice versa*. It follows from the data that *N*_max_ values change robustly in response to changes in the upstream activation parameters but are relatively weakly affected by changes downstream. For example, the Gα *N*_max_ values in Fig 2B do not change in response to modifications in the downstream PIP_2_ breakdown rates and channel sensitivity to DAG; however, they increase as GDP to GTP exchange rate and Gq concentration increase. Moreover, as the concentration of PLC that works as a sink for Gα decreases, the Gα *N*_max_ increases drastically, indicating that at low PLC levels Gα diffusion becomes a limiting factor.

Dependencies of median latencies and amplitudes on the times of *N*_max_ are shown in Fig 2C and 2E, respectively. Again, the character of scatter plots resembled that observed in the correlations between the times of *N*_max_ and latency in the analysis of individual simulations in control (Fig 1F), with random variability increasing with distance of the molecular stage in question from the QB. However, in contrast to the weak positive correlations found between QB amplitudes and the times of *N*_max_ for Gα and Gα-PLC in control (Fig 1E), the correlations between times of *N*_max_ and mean amplitudes after parametric modifications were strong and negative (Fig 2E). The variability in mean half-widths was relatively small and its patterns resembled those for median latency (Fig 2B and 2C) but with less significant correlations.

### QB properties in the model and experiments

#### Variability in QB parameters and their dispersion

In our previous studies of *P*. *americana* photoreceptors we discovered that QB latency dispersion is proportional to the mean latency of the photoreceptor and appears to be a major factor restricting the signaling bandwidth [28]. It was therefore necessary to investigate in the model (1) how latency dispersion depends on the activation parameters of phototransduction and (2) how it relates to another bandwidth-limiting factor, the QB duration.

**Fig 3:**
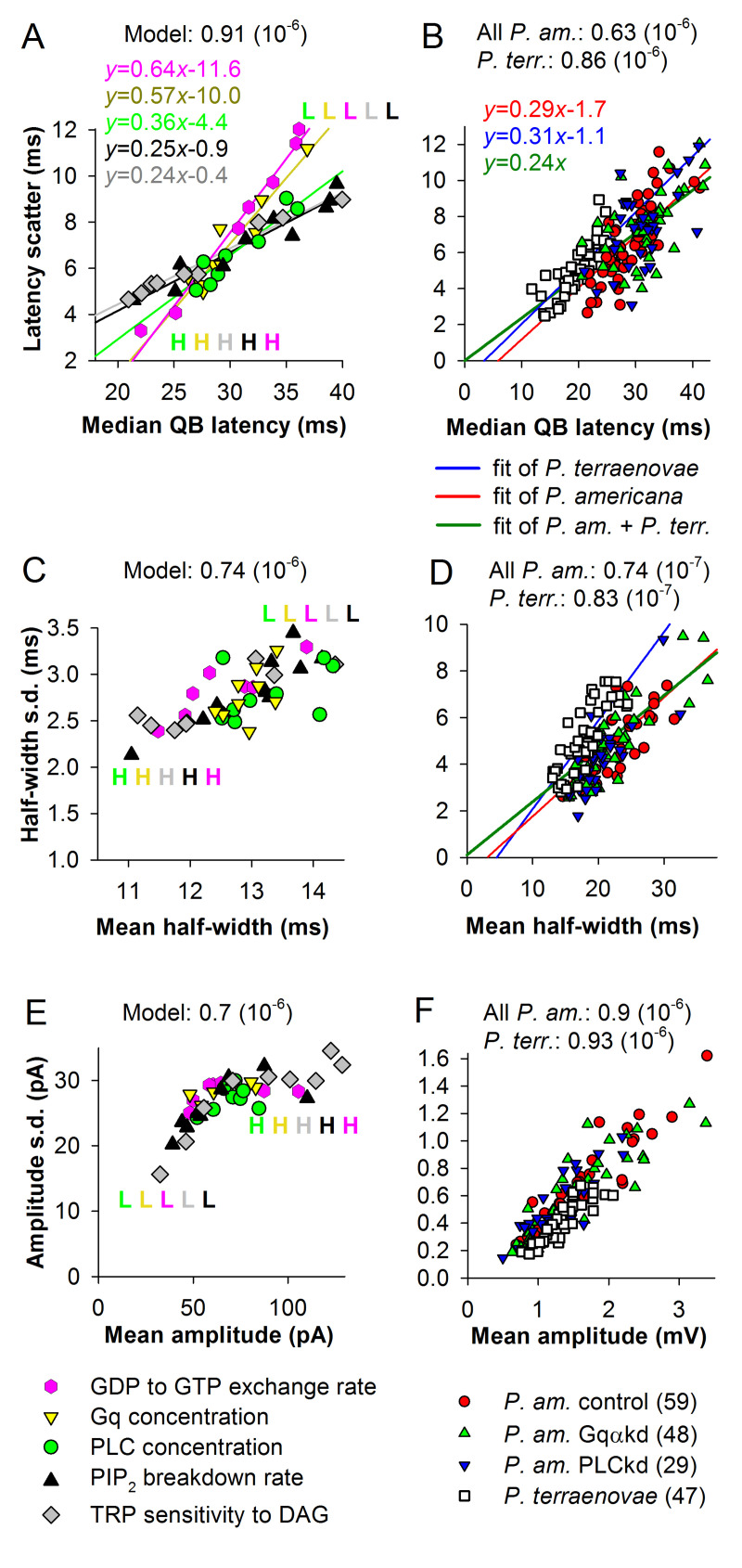
QB latency, half-width and amplitude and their dispersions. (A, B) Correlations between median latencies and latency scatter values for the current bumps obtained in the parametric analysis of activation parameters (A) and for voltage bumps from intracellular recordings from *P*. *americana* and *P*. *terraenovae* (B); data were fitted by linear functions as indicated by the equations and lines of matching colours (in A); in B, the red line is the linear fit of all *P*. *americana* data, the blue line of *P*. *terraenovae* data, and the dark green line of *P*. *americana* and *P*. *terraenovae* data pooled together; in the legends, *n* is the number of cells. (C, D) Correlations between mean QB half-widths and half-width standard deviations for the simulated (C) and experimental (D) data. (E, F) Correlations between mean amplitudes and amplitude standard deviations for the simulated (E) and experimental (F) data.

Fig 3A and 3B shows plots for median QB latency and its scatter for: (1) the results of simulations of *P*. *americana* current bumps involving changes in five activation parameters (Fig 3A); (2) *P*. *americana* voltage bumps obtained in intracellular recording experiments under three conditions (control, Gαkd and PLCkd [29], Fig 3B); and (3) voltage bumps from the blow fly *Protophormia terraenovae* (Fig 3B). Results of the modeling show that: 1) simulations can reproduce the whole range of experimental variability in median QB latency and latency dispersion for *P*. *americana* (and also for *P*. *terraenovae*, with minor modifications); and 2) changes in the upstream cascade activation parameters are associated with stronger changes in latency dispersion than changes downstream.

When the experimental data were fitted by linear equations, the fitting lines (blue for *P*. *terraenovae* and red for all *P*. *americana* data pooled together) crossed the *x*-axis within 6 ms from zero (Fig 3B). However, when all data from the fly and the cockroach were combined and fitted, the fitting line crossed the intercept (thick dark green trace in Fig 3B). This important observation indicates that the variability in latency decreases linearly with median latency. In the model, all lines fitting the individual data sets (Fig 3A) crossed the *x*-axis at >0 ms, with the best approximation to the experimental data achieved for the modifications in the downstream activation parameters (PIP_2_ breakdown rate and TRP sensitivity to DAG).

Dispersions of two other key QB parameters, the half-width and amplitude, were also proportional to the respective mean parameters (Fig 3C–3F). Fig 3C shows a dependence of half-width standard deviations on mean half-widths in the model and Fig 3D in the experiments. All correlations were very strong. Fig 3E shows a dependence of amplitude dispersion on mean amplitude in the model and Fig 3F in the experiments. However, instead of a linear trend as found for QB latencies and half-widths in Fig 3A–3D, Fig 3E demonstrates a saturating function, caused by approaching the maximal number of channels present in the microvillus (25 in these simulations) as the cascade accelerated. This has consequences for the estimation of the amplitude component of phototransduction noise, because fast cascades eliciting large QBs caused by opening of the majority of available channels will be characterized by lower amplitude noise than the relatively slow cascades. However, no evidence of saturation was detected in the experimental data (Fig 3F).

#### Cascade speed and QB duration

Next, we investigated relations between the speed of phototransduction cascade as approximated by median QB latency and other QB parameters.

The QB is the fastest photoreceptor response and thus its duration sets the upper limit of frequencies that a photoreceptor can transfer. But because QBs in a multiphoton impulse response are shifted relative each other according to their latencies, the photoreceptor’s bandwidth must necessarily be influenced by the interaction between QB duration and latency dispersion. The possible scenarios range from a situation when summation of wide bumps characterized by small latency dispersion makes variability in latency inconsequential, to a situation when brief but widely dispersed bumps form a broad response that filters out fast signals.

**Fig 4:**
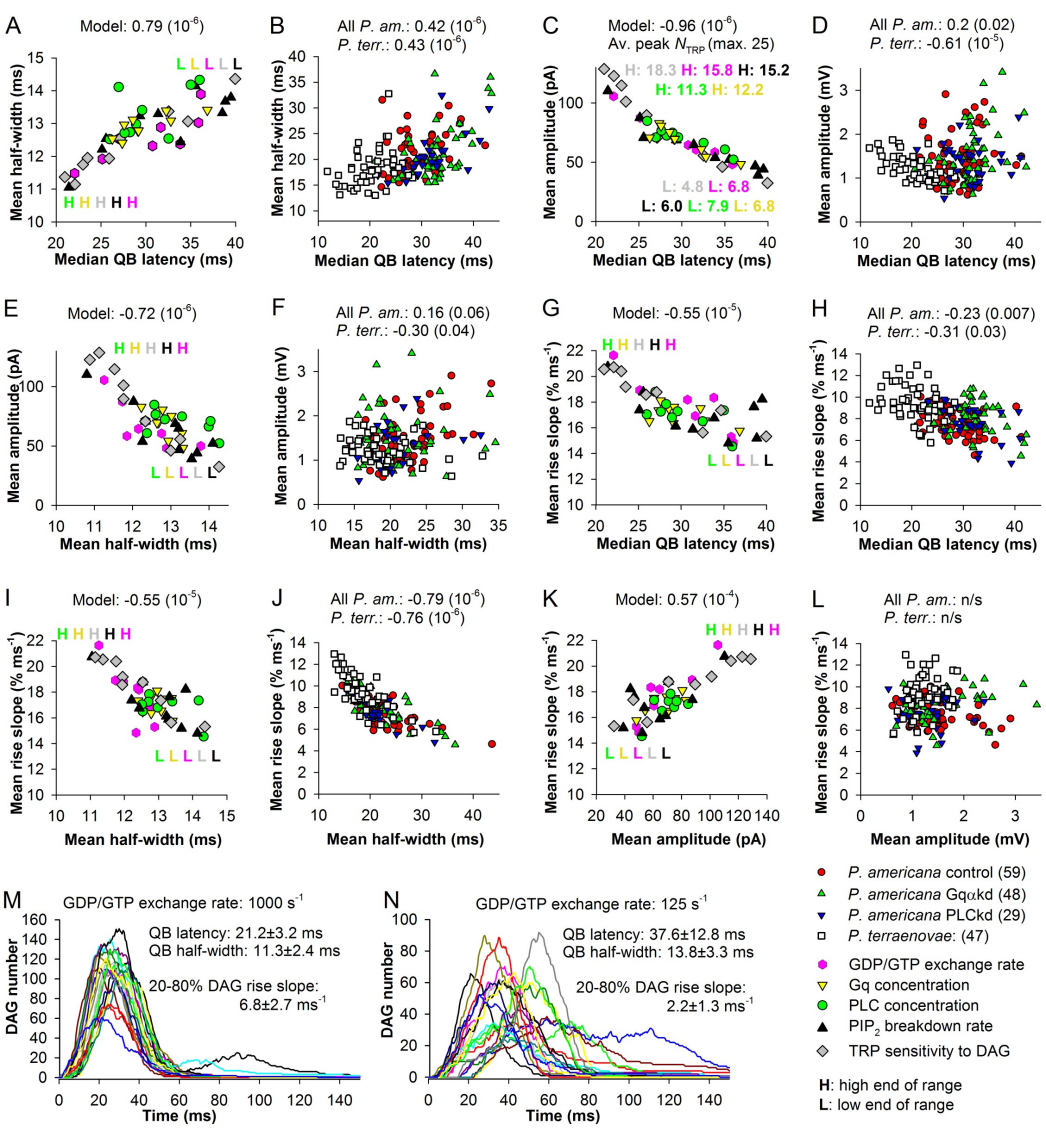
Dependencies of QB properties on the cascade speed and each other. (A, B) Correlations between median latencies and mean half-widths for the simulated current (A) and experimental voltage (B) QB data. (C) Correlations between median QB latencies and amplitudes for the simulated current (C) and experimental voltage (D) QB data; in C, numbers denote average peak numbers of open TRP channels associated with the extreme data points for each activation parameter modification range. (E, F) Correlations between mean QB half-widths and amplitudes for the simulated (E) and experimental (F) data. (G, H) Correlations between median latencies and mean 20–80% QB rise slopes for the simulated current (G) and experimental voltage (H) data. (I, J) Correlations between mean QB half-widths and mean rise slopes for the simulated (I) and experimental (J) data. (K, L) Correlations between mean QB amplitudes and mean rise slopes for the simulated (K) and experimental (L) data. (M, N) Time dependencies of DAG accumulation in a fast (M) and slow (N) cascades; 20 examples are shown in each panel; QB parameters and DAG 20–80% rise slopes are provided in the legends.

Fig 4A shows dependencies of mean QB half-widths on median latencies for the simulated *P*. *americana* current bumps with parameter modification and Fig 4B for the voltage bumps from *P*. *terraenovae* and *P*. *americana*. All correlations were statistically significant (Fig 4A and 4B and Table 1).

**Table 1:** Summary of correlations between statistical properties of QBs obtained in the parametric analysis of the model and electrophysiological experiments. Values are SROCC *ρ*s with *P* given in parentheses. *P*. *americana* correlations are based on the pooled data from control, Gqαkd and PLCkd experiments. Symbols in the column “Fit” indicate the degree of consistency between the correlations in the model and experiments, with the first symbol referring to *P*. *americana* and the second to *P*. *terraenovae* results; “+” stands for the consistent, “0” for the non-significant, and “–” for the significant but opposite correlation. Because latency dispersion correlated with median latency nearly perfectly, no specific correlations between latency scatter and other parameters were included; s.d., standard deviation.

Parameter 1	Parameter 2	Model	*P*. *americana*	*P*. *terraenovae*	Fit
1. Dispersion-related correlations					
amplitude	amplitude s.d.	0.7 (10^−6^) Fig 3E	0.9 (10^−6^) Fig 4F	0.93 (10^−6^) Fig 4F	+ +
half-width	half-width s.d.	0.74 (10^−6^) Fig 3C	0.74 (10^−7^) Fig 3D	0.83 (10^−7^) Fig 3D	+ +
latency	latency scatter	0.91 (10^−6^) Fig 3A	0.63 (10^−6^) Fig 3B	0.86 (10^−6^) Fig 3B	+ +
latency	amplitude s.d.	-0.73 (10^−6^)	0.29 (0.002)	-0.55 (10^−3^)	– +
latency	half-width s.d.	0.90 (10^−6^)	0.33 (10^−3^)	0.5 (10^−3^)	+ +
latency	latency CV	0.55 (10^−4^)	0.34 (10^−3^)	0.42 (0.005)	+ +
latency	amplitude CV	0.9 (10^−6^)	0.24 (0.013)	-0.19 (0.19)	+ 0
latency	half-width CV	0.59 (10^−4^)	0.13 (0.2)	0.38 (0.009)	0 +
2. Correlations between QB parameters					
latency	amplitude	-0.96 (10^−6^) Fig 4C	0.2 (0.02) Fig 4D	-0.61 (10^−5^) Fig 4D	– +
latency	half-width	0.79 (10^−6^) Fig 4A	0.42 (10^−6^) Fig 4B	0.43 (0.002) Fig 4B	+ +
amplitude	half-width	-0.72 (10^−6^) Fig 4E	0.16 (0.06) Fig 4F	-0.30 (0.04) Fig 4F	0 +
latency	rise slope	-0.55 (10^−5^) Fig 4G	-0.23 (0.01) Fig 4H	-0.31 (0.03) Fig 4H	+ +
rise slope	half-width	-0.57 (10^−4^) Fig 4I	-0.79 (10^−6^) Fig 4J	-0.76 (10^−6^) Fig 4J	+ +
rise slope	amplitude	0.57 (10^−4^) Fig 4K	0.06 (0.49) Fig 4L	0.16 (0.26) Fig 4L	0 0

The differences in group-average half-widths between the voltage bumps and the simulated current bumps were due to the low-pass RC-filtering by the membrane of the dark-adapted photoreceptors. The voltage bumps were recorded from cells that varied widely in membrane resistance and capacitance, and this might explain some of the large data spread along the y-axis (Fig 4B). The ranges of latency variation for voltage and simulated current bumps for *P*. *americana* matched well, suggesting that the moderate parameter modification in the model was sufficient to describe the experimental variation in vivo. Regarding a putative mechanism of this dependence, we hypothesized that as smaller latencies are associated with smaller times to peak of DAG concentration and *vice versa* (Fig 2C), the rate of DAG increase in faster photoreceptors must also be higher than in slower cells (Fig 4M and 4N), causing more concerted and synchronous activation of phototransduction channels that would result in narrower bumps.

#### Cascade speed and QB amplitude

A rapid and strong increase in DAG concentration can be expected to activate more transduction channels than a slow increase, and this would manifest in a negative correlation between median QB latencies and amplitudes. We tested this hypothesis in Fig 4C and 4D. In the model, we found a very strong negative correlation between median QB latencies and mean amplitudes (Fig 4C). As illustrated by the values that denote the average peak numbers of open TRP channels associated with the high or low extremes of the activation parameter ranges, high mean amplitudes were caused by opening of more channels at the peak of the QB.

We found an experimental conformation for the relation between mean QB latency and amplitude in the data from *P*. *terraenovae* (Fig 4D). The negative correlation was statistically significant. However, no similar correlation was found in *P*. *americana*, where mean QB amplitudes instead correlated weakly positively with median QB latencies (Fig 4D). While these findings could reflect genuine differences between the phototransduction cascades of the two dissimilar species, the weak cockroach correlation was unreliable and possibly caused by high intrinsic variability between the photoreceptors [32].

Because mean QB half-widths correlated positively with median latencies, we also tested the correlations between amplitudes and half-widths. A strong correlation was found in the model (Fig 4E) and a weak correlation in the experimental *P*. *terraenovae* data (Fig 4F).

#### Cascade speed and onset kinetics

We hypothesized that changes in the rate of DAG increase (Fig 4M and 4N) could be linked to changes in the TRP activation rate, which would manifest in a negative correlation between the latencies and QB onset rates. This hypothesis was supported both in the model and experiments (Fig 4G and 4H and Table 1). Fig 4G and 4H show statistically significant negative correlations between median QB latencies and onset rates measured as normalized 20–80% rise slopes. Consistent with the above-described correlations between latencies, amplitudes and half-widths, the rise slopes also correlated negatively with mean half-widths (Fig 4I and 4J), and positively with amplitudes in the model (Fig 4K) but not experiments (Fig 4L).

Table 1 summarizes our analysis of QB properties. Correlation coefficients are provided for various pairs of QB parameters and their dispersion measures, with the emphasis on the dependencies on the cascade speed. Because dispersions of QB latencies correlated very strongly positively with median QB latencies, we did not give the almost duplicate correlations between the latency scatter and other QB parameters. It should be understood that whenever a statistical QB property correlates significantly with the mean or median QB latency, it also similarly correlates with a measure of latency dispersion (the standard deviation or scatter values). The correlations in Table 1 show that cell-to-cell variabilities in the main QB properties found in the experiments or cascade-to-cascade variabilities in the model correlate with each other and are *largely dependent on the speed of phototransduction*. The overall best fit with the model predictions was achieved for *P*. *terraenovae*.

### QB properties and signaling bandwidth

In the previous sections we investigated the properties of single QBs in experiments and in the model. However, any multiphoton impulse response is a superposition of multiple bumps shifted relative each other due to variations in latency. In the following, we refer to the experimentally obtained responses to flash stimulation (here 1 ms pulses) as impulse responses, while calling their simulated analogs the “composite” responses. To obtain average bumps, we carefully aligned the rising phases of individual bumps (at ~10% of bump amplitudes) and then averaged. Because of latency dispersion, an impulse response is always wider and has a slower onset than the average QB, causing additional low-pass filtering of the signal.

As we showed above, the QB latency dispersion, mean half-width and onset kinetics can be lowered by speeding up the cascade. In vivo, differences in the cascade speed are observed between insect species with dissimilar compound eye morphologies and visual ecologies [10], and they can manifest in two forms, as the differences in QB latency distributions between the dark-adapted photoreceptors (Fig 3B), and in the character of cascade acceleration during light adaptation or absence thereof [10]. Therefore, given the multifaceted effects of the cascade speed on QB properties, it was important to investigate how it influences the signaling bandwidth.

### QB duration, latency dispersion and signaling bandwidth

**Fig 5:**
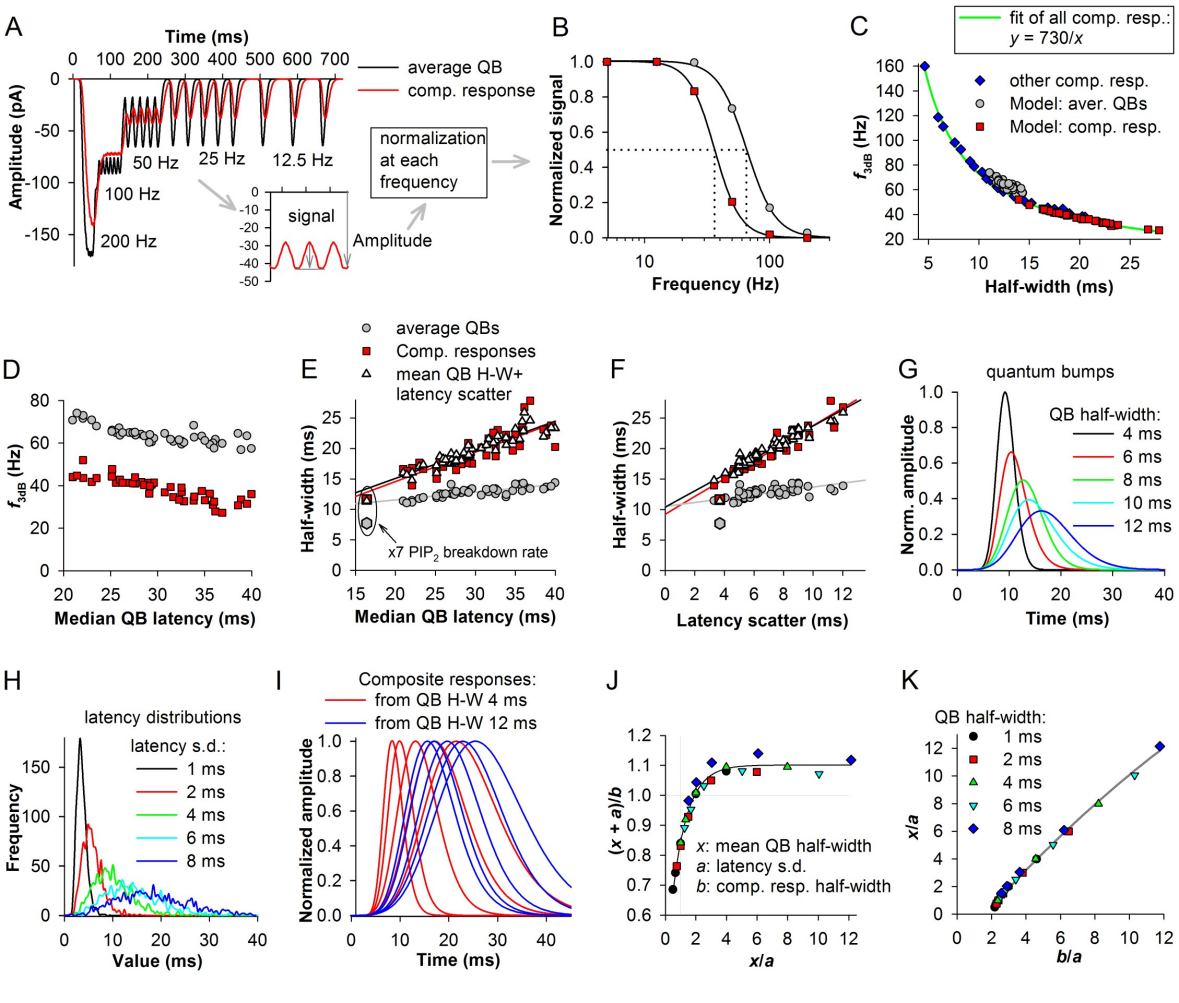
QB statistics and signaling bandwidth. (A) Examples of simulated flickering-frequency response functions constructed by summation of lognormal fits of average bumps and composite responses at different frequencies of stimulation; QB sets were from the parametric analysis; composite responses were obtained by averaging all bumps evoked during the 200-trial runs of the model as they appeared, without alignment. Subtracting the local minima from the maxima and then dividing by the amplitude at each frequency as indicated in inset gave the signal amplitudes. (B) Dependencies of signal amplitudes from A on frequency; plots were fitted by a Hill equation; dotted lines denote corner frequencies (*f*_3dB_). (C) Dependencies of corner frequencies on half-widths of average bumps and composite responses; blue diamonds represent 25 composite responses obtained in separate simulations, with ten examples shown in I. (D) Dependencies of corner frequencies on median QB latencies. (E) Plots of half-widths against median QB latencies; white triangles are the sums of average QB half-widths with the corresponding latency scatter values; regression lines are plotted for composite responses (red), sums of average QB half-widths and latency scatter values (black) and average QB half-widths (gray); data set in the ellipse represents a cascade characterized by a seven-fold acceleration of PIP_2_ breakdown rate (not included into the fitted set). (F) Plots of average QB or composite response half-widths against latency scatters. (G-K) Investigation of the dependence of the composite response half-width on the QB half-width and latency dispersion. (G) Five QB-like lognormal waveforms. (H) Five latency distributions comprised of 1000 values each. (I) Two sets of composite responses constructed by combining the QBs from G with five latency distributions from H. (J) A relation between the sum of QB half-width and latency standard deviation divided by the half-width of the corresponding composite response and the ratio of QB half-width to latency standard deviation; data were fitted with an exponential rise to maximum equation; straight grey lines provide reference. (K) A rearranged relation suitable for easy extraction of the QB half-width from the composite responses to an instantaneous stimulus when latency standard deviation is known.

In the parametric analysis above we obtained many QB sets characterized by different latency, duration and amplitude distributions (Figs 3 and 4). Fig 5 demonstrates that the composite responses produced by averaging the QB sets also vary in their duration and kinetics. How can these differences affect the signal transfer in the absence of other variabilities and sources of noise? To investigate, we modeled the frequency response functions using the average QBs and the associated composite responses (Fig 5A). Although both the average QBs and composite responses were based on of >150 traces, and the residual variability arising from the cascade stochasticity was small, it was still not negligible, especially when slower cascades were concerned. Therefore, we fitted the waveforms using a lognormal function and used the fitted curves to estimate noise-free frequency response functions.

The functions were constructed in the following way. The same traces were shifted relative to each other by 5, 10, 20, 40, 80, or 200 ms (corresponding to frequencies of 200, 100, 50, 25, 12.5 and 5 Hz) and summed, imitating responses to series of instantaneous flashes of incrementing frequency (a flickering stimulus). Then a signal was obtained at each frequency by subtracting the minimal amplitude from the maximal one and dividing the difference by the maximal absolute amplitude (Fig 5A inset). The signals were then plotted against frequency. Such frequency-dependencies are usually best fitted by a Hill equation. The examples in Fig 5A and 5B show modeled flickering-frequency response functions for the average QB in control and the associated composite response, with the dotted drop lines denoting 3 dB corner frequencies (*f*_3dB_).

We obtained corner frequencies for 46 average QB-composite response pairs from the parametric analysis. Corner frequencies plotted against half-widths of average QBs and composite responses revealed a non-linear correlation (Fig 5C). To explore a broader range of the dependence of the corner frequency on the characteristic duration of the response, we constructed a number of composite responses by using arbitrary QBs and latency distributions (Fig 5G–5I), determined the corner frequencies and introduced the data to the plot (blue diamonds). Consistently with the previous theoretical predictions [5], the best fit of all composite responses was achieved using a first-order reciprocal equation *y* = 730/*x* (Fig 5C).

Corner frequencies associated with the data from the parametric analysis simulations also correlated strongly negatively with median QB latency (Fig 5D, indicating that slow phototransduction cascades characterized by high latency dispersion can poorly transmit higher frequencies, and *vice versa*.

Importantly, we found that the composite response half-width in the model can be reliably approximated by summing the half-widths of the average QB with the latency scatter values of the associated median latencies (Fig 5E and 5F). The red, black and gray regression lines in Fig 5E and 5F describe, correspondingly, the composite responses, the sums of average QB half-widths with the latency scatter values, and the average QBs. All lines crossed the *y*-axis at ~10 ms, denoting the lower limit of both the QB and composite response durations for the simulated *P*. *americana* phototransduction cascade in the absence of modifications to QB termination.

It should be noted that because the latency scatter estimate was very similar value-wise to the latency standard deviation, the two could be used interchangeably to predict the mean QB half-widths from impulse responses and *vice versa*. For instance, since calculation of the latency scatter depends on a lognormal fitting of the latency distribution, the scatter measure can be used when the sample is large, consisting of many hundred data points. Otherwise, standard deviation might be a measure of choice.

Because developing a method to extract statistical information about QBs directly from impulse responses is important, we tested in separate simulations how the composite response duration is affected by both mean QB half-widths and latency scatter. We used five QB-like waveforms, from a very narrow with a half-width of 4 ms to a comparatively wide with a half-width of 12 ms (Fig 5G) together with five latency distributions (Fig 5H). By combining all QBs with all latency distributions, we generated 25 composite responses, with ten examples shown in Fig 5I. The corner frequencies associated with these composite responses are shown in Fig 5C (blue diamonds).

To investigate the relations between the QB half-width and latency standard deviation, on the one hand, and the composite response half-width, on the other hand, we plotted the ratios of the predicted composite response half-width (the sum of QB half-width “*x*” and latency standard deviation “*a*”) to the actual composite response half-width (“*b*”) against the ratios of QB half-width to latency standard deviation *x*/*a* (Fig 5J). The non-linear saturating dependence indicates that when latency dispersion is large compared to QB half-width, their sum progressively underestimates the composite response half-width. However, when the *x*/*a* ≥ 4, the ratio (*x* + *a*)/*b* becomes constant. By plotting *x*/*a* against *b*/*a* and then fitting the dependence with a polynomial equation (Fig 5K), it is possible to determine the mean QB half-width when both the impulse response half-width and latency dispersion are known. This method was used below to estimate QB properties from experimental impulse responses.

### Comparison to experimental data

**Fig 6:**
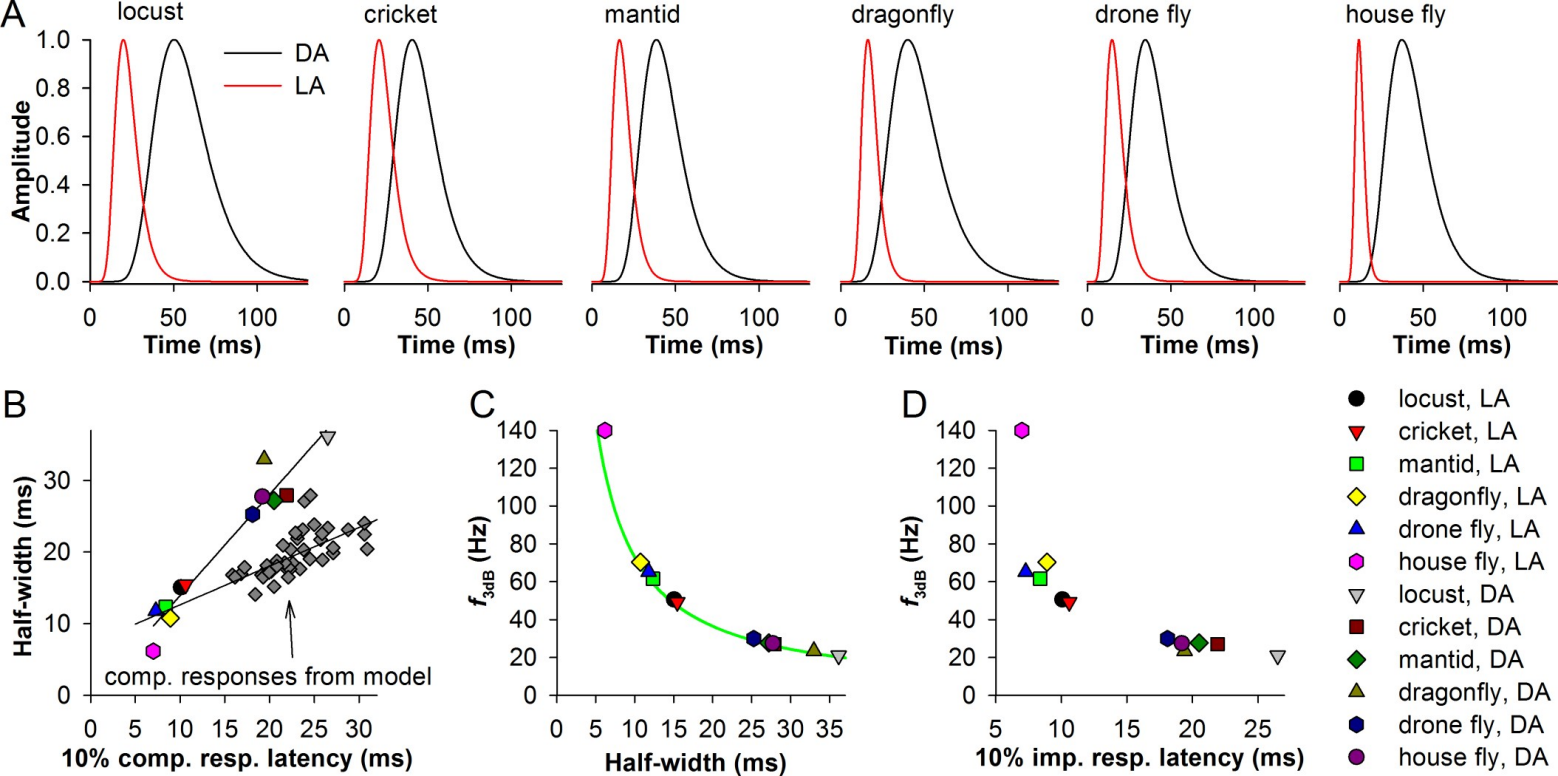
Comparison of impulse responses in different insect species. (A) Reconstructed average impulse responses of light- and dark-adapted (LA and DA, respectively) photoreceptors from six species: locust *Locusta migratoria*, bush cricket *Tympanophora pellucida*, praying mantis *Tenodera australasiae*, dragonfly *Hemianax papuenis*, drone fly *Eristalis tenax*, house fly *Musca domestica*, as indicated; in the original study [10] the waveforms were fitted by lognormal functions, the parameters of which we used for the reconstructions. (B) Plots of half-widths of *in vivo* voltage impulse responses and *in silico* composite responses comprised of current bumps against the response latencies determined at the 10% amplitude level; the data were fitted by linear functions. (C, D) Dependencies of the corner frequency on the half-width (C) and 10% response latency (D) of the impulse responses; in C, the same reciprocal equation *y* = 730/*x* was used as in Fig 5C.

Thus far, we found a suite of several QB properties needed for transmission of higher frequencies by composite responses, including small median (or mean) latency, low latency dispersion and small half-width. As we showed above, these properties all depend on the speed of phototransduction. How do the results of our QB experiments and simulations correspond to the experimental impulse responses? For a test, we used average impulse responses triggered by 1 ms flashes in light- and dark-adapted photoreceptors from six insect species [10]. The waveforms of average impulse responses were reconstructed using the lognormal fitting parameters provided in the article. Fig 6A shows six pairs of impulse responses, all showing drastic changes in the timing, duration and kinetics as a result of light adaptation. We then obtained their 10% latencies, half-widths and estimated the corner frequencies as in Fig 5A and 5B.

Fig 6B shows the half-widths of the experimental impulse responses and the half-widths of the composite responses from our parametric simulations plotted against their latencies. The group differences visualized with the linear fitting curves are due to membrane filtering, which is absent in the current QB-based simulated composite responses. Membrane filtering strongly expands the impulse responses of the dark- but not light-adapted photoreceptors because membrane resistance is usually much higher in the dark- than a light-adapted photoreceptor state. Similar differences between the in-silico current and in-vivo voltage bumps can be seen by comparing Fig 4A and 4B. Therefore, the observed differences in the impulse response durations can only partly be attributed to the differences in the cascade speed, with the rest accounted by dissimilarities in the extent of membrane filtering and QB termination kinetics.

Correlations between the impulse response half-width and the corner frequency (Fig 6C) and between the impulse response latency and the corner frequency (Fig 6D) are consistent with the composite response modeling in Fig 5. It should be noted that although the dependence of the signaling bandwidth and thus information capacity on the impulse response duration is well-known [5], their mechanistic connections to the cascade speed have not been studied in the past.

### Transduction and photon noises

In the previous section, we investigated the limits imposed on one aspect of signal transfer, the bandwidth, by differences in the speed of phototransduction between cascades in the simulations. However, the information-carrying capacity of a channel under the assumption of linearity of input and Gaussian noise depends on both the bandwidth and signal-to-noise ratio [3]. Therefore we next investigated how the intrinsic variabilities in QB properties generate noise and how this noise can alter signal transfer under various conditions.

### Variabilities in QB properties

The parametric space of our variability analysis was narrowed by the finding that all main QB properties are interlinked when one compares average bumps elicited by different cells in experiments or by different cascades in the model. Thus we showed that the mean QB half-width and its dispersion increased with the rising latency (Fig 4A and 4B and Table 1), that the mean QB amplitude increased with the decreasing latency (Fig 4C and 4D and Table 1), and that the mean QB amplitude and half-width correlated negatively (Fig 4E and Table 1). Changes in median QB latencies, mean half-widths and amplitudes were accompanied by linear changes in the associated dispersions (Fig 3), with the exception of mean QB amplitude in the model where a saturating function was found (Fig 3E).

There is an important distinction between variabilities in QB amplitudes and half-widths, on one hand, and variability in latencies, on the other hand: while the first two are measures of the actual QB properties, the latter is a measure of QB timing. Thus, while the noises introduced by variabilities in amplitudes and half-widths were proportional to the respective coefficients of variation (CVs), the noise caused by the variability in latency was proportional to latency standard deviation itself. Dependencies of all CVs on the speed of phototransduction are presented in Table 1. Out of nine correlations (three for the model and six for the experiments), seven were statistically significant and positive, indicating that *all measures of QB noise increase when the cascade slows down and vice versa*.

### Signal-to-noise ratios

**Fig 7:**
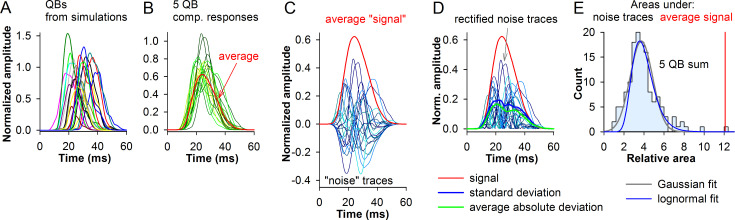
Construction of noise-containing composite responses. (A) 20 simulated control *P*. *americana* QBs with variable amplitudes, latencies and durations; QBs were divided by the average bump amplitude (-70.8 pA). (B) 20 composite responses (thin traces in hues of green) each comprised of 5 randomly selected QBs and their average (thick red trace); the traces were divided by the amplitude of the set average. (C) The average “signal” was subtracted from each of the traces from B yielding “noise” traces (thin traces in hues of blue). (D) The noise traces were reconstructed by taking absolute values from B; thick green line is the average absolute deviation; the thick blue trace is the standard deviation. (E) Distribution of areas under the noise curves from D; red line indicates the area under the average composite response. The noise distribution was fit with a normal and lognormal equations as indicated.

To investigate how the noise emitted by the phototransduction cascade affects photoreceptor signaling, we had to first develop a measure of SNR. The methodology used to create noise-containing composite responses is illustrated in Fig 7.

First, we constructed composite responses by summing QBs randomly selected from a simulated control *P*. *americana* QB set (Fig 7A). Several composite responses consisting of 5 QBs with variable latencies, amplitudes and half-widths are shown in hues of green in Fig 7B, with the thick red trace representing the average of 120 such responses. By subtracting the average “signal” trace from each of the original composite responses, a set of noise traces could be obtained (Fig 7C, in hues of blue). There are several approaches to estimate the SNR of a composite response. These include using the ratios of the signal peak to either the peak of the standard deviation or average absolute deviation traces (Fig 7D), or the ratios of the areas under the signal and noise curves. We considered the ratio of areas to be a more robust measure than the ratio of peak amplitudes because the area under the average “signal” curve is invariant to the waveform stretching or shortening. However, before using the parametric statistics, its applicability had to be tested. The distribution of absolute areas under the individual rectified noise traces (Fig 7E) was fitted with both Gaussian and lognormal equations, revealing a normal-like distribution. We therefore selected the area under the standard deviation curve to serve as a convenient measure of noise (Fig 7D). Because each SNR estimate was based on about 100 composite responses, the SNR was not very sensitive to the influence of outliers.

### Transduction noise in the normal *P*. *americana* cascade

**Fig 8:**
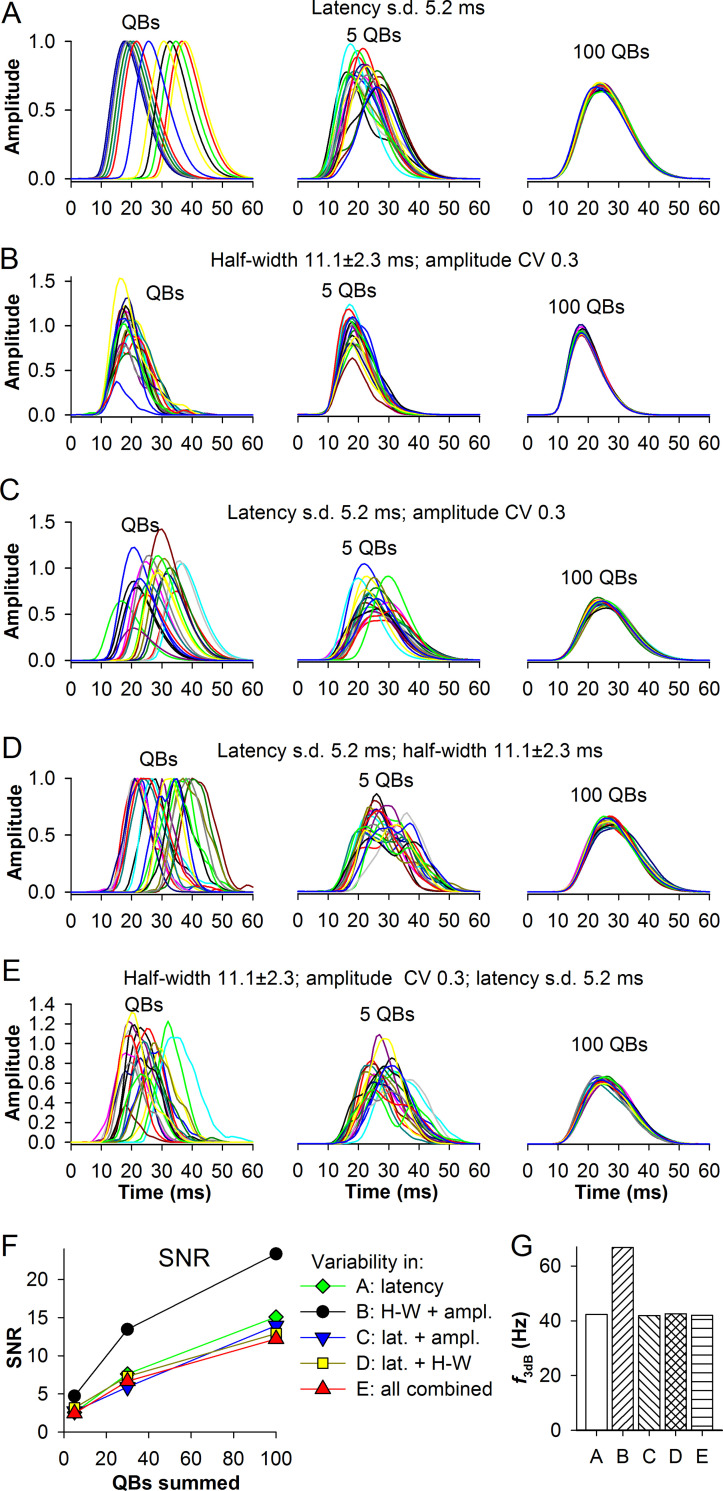
Transduction noise in the dark-adapted *P*. *americana* cascade. SNR functions were constructed to study the separate components of the transduction noise associated with the normal modeled *P*. *americana* current QBs and their combinations. A through E: examples of QBs are shown to the left, composite responses comprised of 5 QBs in the center and comprised of 100 QBs to the right; normalized amplitudes were derived: for QBs, by dividing values in each group by the amplitude of the group-average trace, and for the composite responses, by additionally dividing the obtained QB sums by 5 or 100, respectively. (A) Effects of variation in latency; to produce composite responses, the average QBs were shifted relative to each other and then summed. (B) Effects of variation in QB half-widths *and* amplitudes; aligned individual QBs from the modeled set were used. (C) Effects of variation in QB latencies *and* amplitudes; average QBs were multiplied by the amplitudes and shifted by latency values from the modeled set. (D) Effects of variation in QB latencies *and* half-widths. (E) Effects of the combined variations in QB latencies, amplitudes and half-widths. (F) Dependencies of the maximal SNRs on the QB summation level for different combinations of QB variabilities. (G) Corner frequencies at 100 QB summation level obtained using the fitting curve in Fig 5C; letters in the *x*-axis tick label refer to the panels of this figure; H-W, half-width.

First, we tested how the transduction noise alters signaling accuracy by using the simulated *P*. *americana* control QBs (Fig 8). Fig 8A–8D compares the effects of several combinations of QB variabilities on the composite responses as indicated (with the exception of separate variabilities in half-widths and amplitudes). The left subpanels show QBs before their random recombination into composite responses consisting of 5 and 100 QBs. Fig 8E shows the effects of all three QB variabilities combined. Fig 8F shows dependencies of SNR on the number of QBs in the response and Fig 8G the corner frequencies at the 100 QB summation level (estimated using the fitting equation in Fig 5C). It can be seen that introduction of latency dispersion with a standard deviation of 5.2 ms strongly reduced both SNR and corner frequencies. The effect of variability in latency alone on SNR appeared to be stronger than the effects of variabilities in QB half-width and amplitude combined (Fig 8F). When in addition to latency, either half-width or amplitude, or both, were varied, SNR decreased further by <20% (Fig 8C–8F). While this could suggest that the variabilities in QB amplitudes and half-widths contribute relatively little to the overall noise, the lack of substantial differences in SNR between different combinations of variabilities was in fact due to non-additivity and complex mutual dependencies of the three noise sources as we show below.

### Quantum bump statistics from impulse responses

One of the goals of this work was to determine how adaptive changes in the phototransduction cascade alter the photoreceptor signaling. However, such analysis requires comprehensive information about the statistical properties of QBs elicited by the light-adapted cascade, which cannot be obtained directly because individual light-adapted QBs are too small to be measured.

In the previously published analysis, the average shapes and statistics of QBs were derived from the macroscopic responses [9]. However, the estimated QB shapes were not lognormal as they should be, and, crucially, the accompanying latency distributions were stationary, indicating that the cascade did not accelerate with light adaptation. Such findings in the highly-visual *D*. *melanogaster* are not only counterintuitive but also inconsistent with the still earlier reported differences between impulse responses of dark- and light-adapted photoreceptors of dissimilar species (Fig 6A), which point to a dramatic acceleration of both the QB onset and termination in the light-adapted state [10].

To estimate the QB statistics from the impulse response data for different species and under different adaptation states, we performed a five-stage analysis by using the correlations between the QB and composite response parameters discovered and described above.

**Fig 9:**
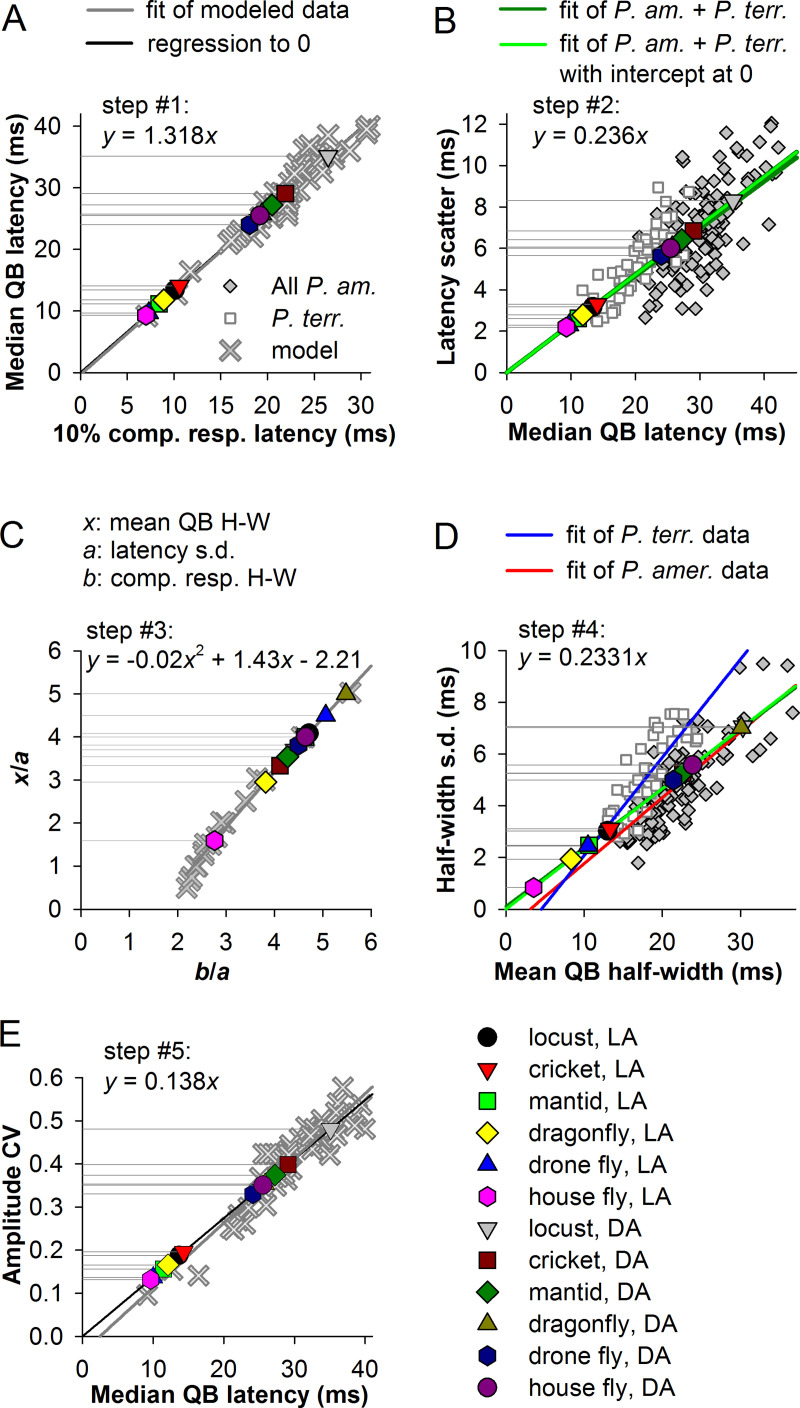
Extraction of QB statistics from impulse responses. (A) Step 1: determining median latencies from the latencies of composite or impulse responses. Equations in the figure describe the curves used in the estimation of QB parameters. Correlation between median QB latencies and 10% latencies of the composite responses for the modeled data was fitted by two linear functions, one without (thick gray trace) and another (black) with intercept fixed at 0. To cover a larger range, in addition to the previously computed single-parameter modification QB sets, we generated two more sets of QBs by combining stimulating modifications of several activation parameters. These QB sets were characterized by small median latencies (crosses under the LA house fly and cricket data points). Median QB latencies describing the QB sets underlying the in-vivo impulse responses from Fig 6A were derived by plotting the 10% impulse response latencies over the black trend line; the *y*-axis drop lines denote the resulting median QB latencies. In addition to median QB latencies we similarly obtained mean latencies. (B) Step 2: determining the latency scatter values. By using the correlations between the median QB latencies and the latency scatter values from all in-vivo data pooled together, we similarly derived QB latency scatter values for the impulse response data; here and in D, the dark green line denotes the regression of the combined cockroach and fly data and the green line of the combined cockroach and fly data with intercept fixed. Latency standard deviation values were obtained using a nearly identical correlation between mean QB latencies and latency standard deviations. (C) Step 3: determining mean QB half-widths from the relationships established in Fig 5K by using the polynomial fitting curve as indicated; notice that *x* in the equation stands for *b*/*a*. (D) Step 4: determining half-width standard deviations by using the regressions for the combined cockroach and fly experimental data as in B. The thick red line denotes the regression for *P*. *americana* data, the blue line for *P*. *terraenovae* data. (E) Step 5: obtaining amplitude CV values from the modeled data; a similar procedure as in A was used; color coding as in A.

First, we predicted median QB latencies from the impulse responses. We plotted the 10% latencies of the composite responses from our parametric simulations against the median latencies of the constituent QBs. Fig 9A shows the resulting linear correlation, which we fitted by a linear equation (thick gray line). The intercept was almost exactly at zero, as would be expected because in the absence of QB latency dispersion the composite response approximates the average of the aligned constituent QBs. Then we positioned the 10% latencies of the experimental impulse responses from Fig 6 on the fitting line and obtained the expected median latencies for the QB sets comprising the impulse responses.

Next, we obtained the latency dispersion estimates from the linear dependencies of latency scatter on the median latency (Fig 3A and 3B) by pooling together all available experimental data and fitting them by a linear function (Fig 9B). In the same way we also obtained the mean QB latencies and latency standard deviations, the latter nearly identical to the latency scatter values.

Then we used the derived latency standard deviation values and the half-widths of the experimental impulse responses to estimate mean QB half-widths (Fig 9C). We used the empirical relation obtained during modeling of QB summation into composite responses (Fig 5G–5K).

At the fourth stage, we estimated the half-width dispersions in terms of standard deviations (Fig 9D). Again, we had two distinct sets of experimental data characterized by dissimilar regression trend lines. However, when all in-vivo data were pooled together and fitted, the resulting regression line (dark green) coincided nearly perfectly with the fitting line fixed at the origin (green).

Finally, using the correlation between median QB latencies and amplitude CVs from the simulations, we estimated the amplitude CVs (Fig 9E). All equations describing the fitting lines used in these approximations are given in the corresponding panels.

### Transduction noise, photon noise and light adaptation in the house fly and locust

**Fig 10:**
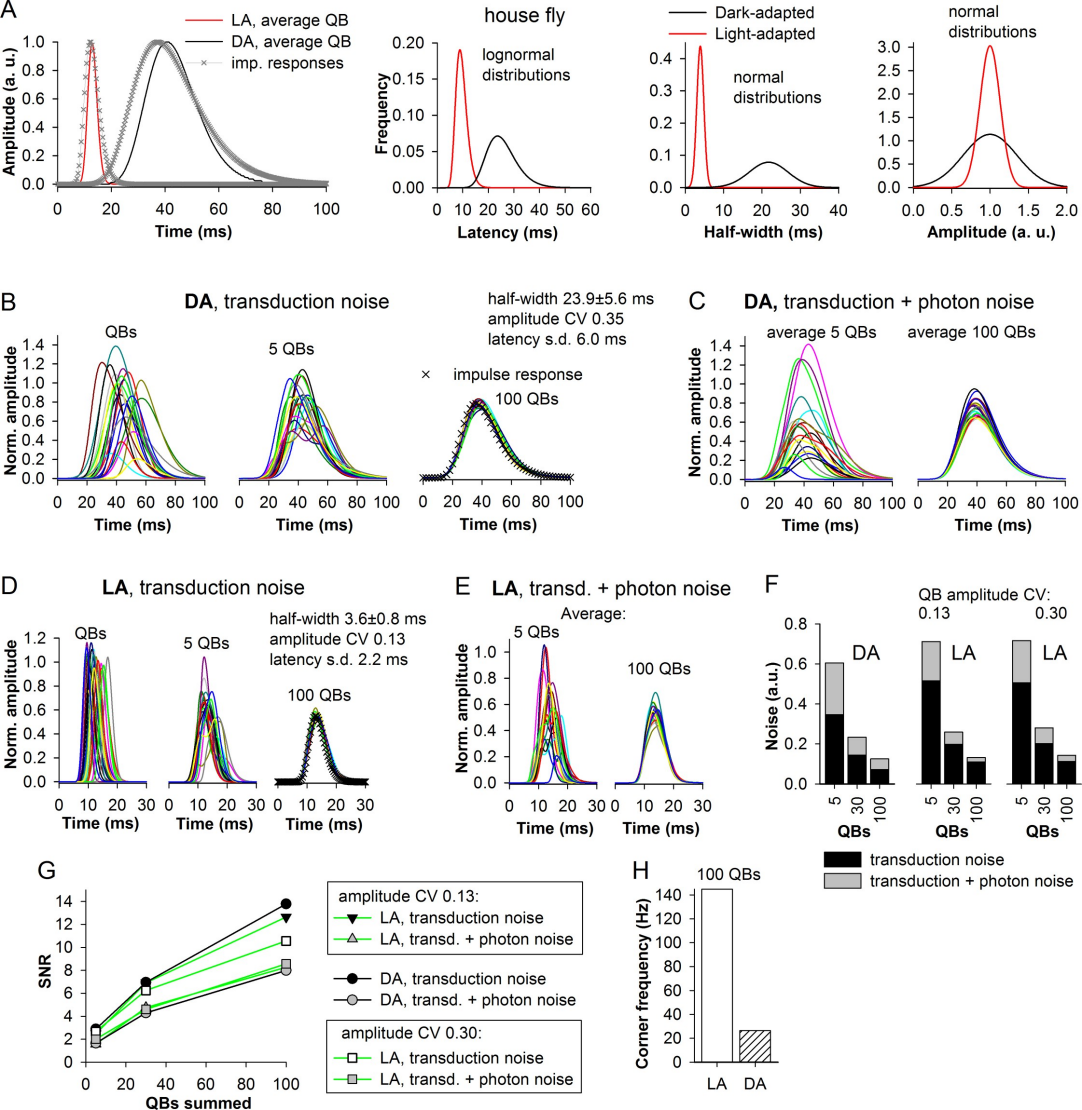
Transduction and photon noise in the house fly. (A) To the right: lognormal fits of the experimental impulse responses from the house fly (Fig 6A) and the estimated mean QBs obtained with the algorithm presented in Fig 9; the timing of the impulse response traces is authentic; the average QBs were placed so that their 10% latencies were correct. Center-left through right sub-panels: the distributions of derived QB latencies, half-widths and normalized amplitudes describing the dark- and light-adapted house fly *Musca domestica* photoreceptors. (B, D) Left subpanels: examples of reconstructed dark- (B) and light-adapted (D) house fly QBs. Center subpanels: random sums of 5 QBs. Right subpanels: random sums of 100 QBs fitted by the original house fly impulse response from A (crosses); all composite responses in this figure and Fig 11 were additionally divided by the set-average amplitudes for presentation purposes. (C, E) The same as in B and E but with random variation in the number of QBs in each composite response in accordance with Poisson distributions characterized by appropriate means (5 and 100, and also 30 for data presented in F and G); notice differences in time scales between B and C, and D and E. (F) Three sets of normalized noise values associated with a dark- and light-adapted phototransduction cascades, without (black) and with photon (gray) noise at different summation levels constructed using the composite responses from B, C, D and E; notice that we compared not the absolute but relative levels of noise because QBs evoked in the dark-adapted photoreceptor are much bigger than the light-adapted QBs, and so the noise is proportionally higher; therefore, the noise values in F (arbitrary units, a.u.) are simply reciprocals of SNR. (G) Dependencies of SNR on the QB summation level under different conditions. (H) Corner frequencies associated with dark- and light-adapted photoreceptors at 100 QB summation level obtained using the fitting curve in Fig 5C.

**Fig 11:**
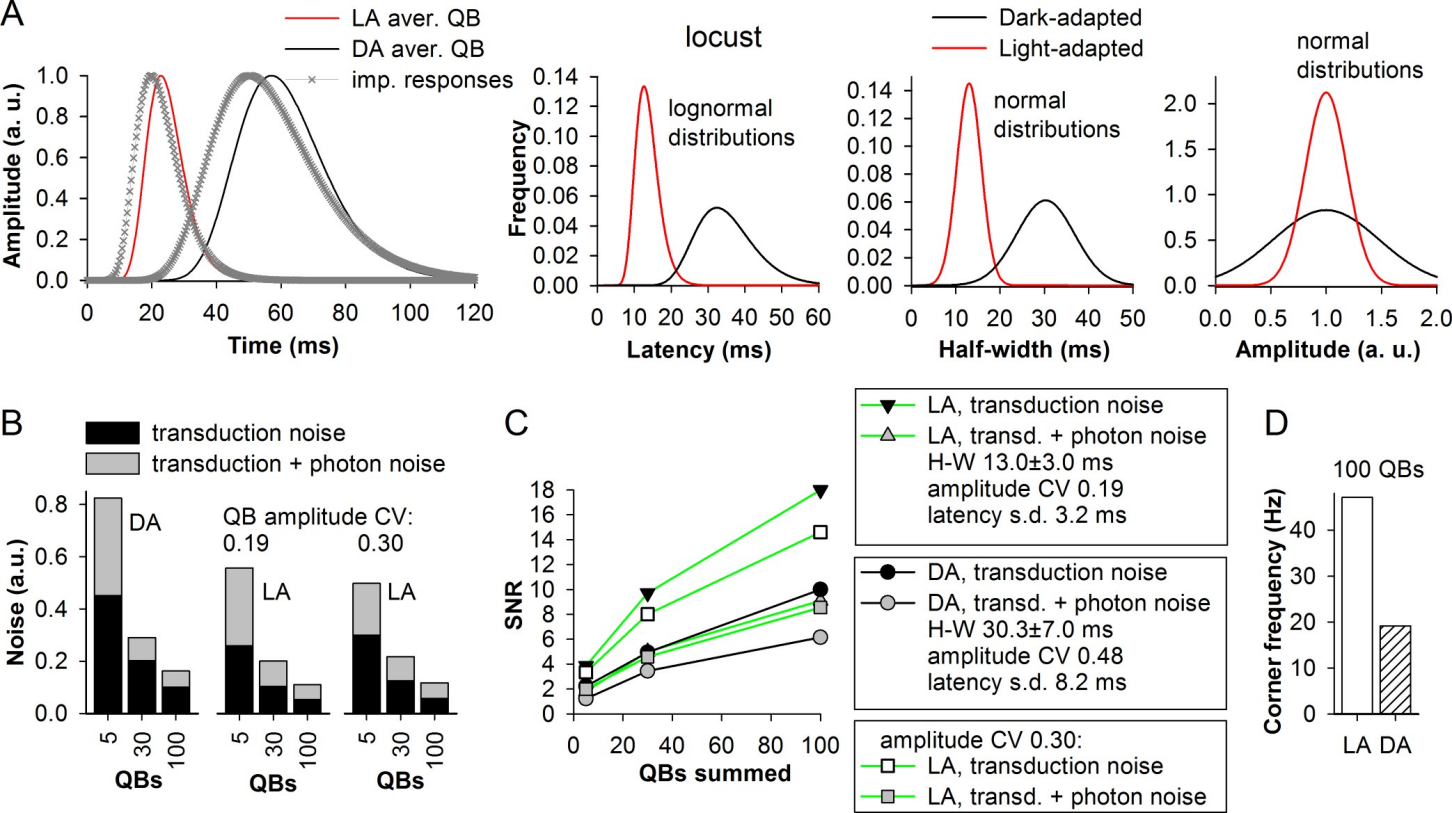
Transduction and photon noise in the locust. (A) To the right: lognormal fits of experimental impulse responses from the locust together with the estimated mean QBs. Center-left through right sub-panels: distributions of derived QB latencies, half-widths and normalized amplitudes for the dark- and light-adapted locust *Locusta migratoria* photoreceptors. (B) Three sets of noise values associated with a dark- and light-adapted phototransduction cascades, without and with photon noise at different summation levels; as in Fig 10F the values are reciprocals of SNR. (C) Dependencies of SNR on the QB summation level under different conditions. (D) Corner frequencies associated with dark- and light-adapted photoreceptors.

Figs 10A and 11A show the estimated mean QB half-widths (left, relative to the duration of the associated impulse responses), latency distributions (center-left), half-width distributions (center-right) and normalized amplitude distributions (right) for the dark- and light-adapted photoreceptors of the house fly and locust. The house fly represents the most visually-guided species characterized by the fastest phototransduction in our sample, whereas the locust is the slowest species. The left sub-panels of Figs 10A and 11A indicate that the impulse response durations are mainly determined by the average QB duration. Importantly, all three transduction-related variabilities decreased significantly with light adaptation (Figs 10A and 11A, three right sub-panels). If latency dispersion would not decrease during light adaptation, it can be expected to expand the impulse responses of the light-adapted photoreceptor to a much greater extent than it was observed.

The derived QB parameters were used to evaluate transduction noise generated by the dark- and light-adapted photoreceptors of the house fly and locust (Figs 10B–10G, 11B and 11C). Fig 10B and 10D shows examples of QBs and composite responses describing responses of dark- (Fig 10B) and light-adapted (Fig 10D) house fly photoreceptors, respectively (notice time scale differences). Right sub-panels demonstrate composite responses comprised of 100 QBs, which match well the original impulse response (crosses). The variability between the responses caused by the differences in the transduction noise level decreased as the number of QBs in composite responses increased.

To model the effects of the photon noise, we constructed three Poisson distributions with means of 5, 30 and 100 and then randomly picked from them values to be used as the number of QBs in individual composite responses. For example, instead of the fixed 100 QBs as the composite responses in right sub-panels of Fig 10B and 10D, the new composite responses contained variable numbers of QBs: 107, 103, 92, 105, 90, 97… etc. (Fig 10C and 10E). Addition of photon noise strongly increased the variability in composite responses and decreased SNR. Figs 10F and 11B show reciprocals of SNR values from Fig 10G, which thus denote *relative noise levels*. (Absolute noise levels were not compared because dark-adapted QBs are much bigger than the light-adapted QBs, and so the noise is proportionally higher.) Notice that in these simulations we used two QB amplitude variability coefficients for each light-adapted cascade, one from the model (0.13 in Fig 10F and 0.19 in Fig 11B) and another (0.3) equaling the group-average amplitude CV of *P*. *terraenovae* bumps. This was done to account for the discrepancy between the model and experiments in regard to the dependency of amplitude CV on QB latency (Table 1).

**Fig 12:**
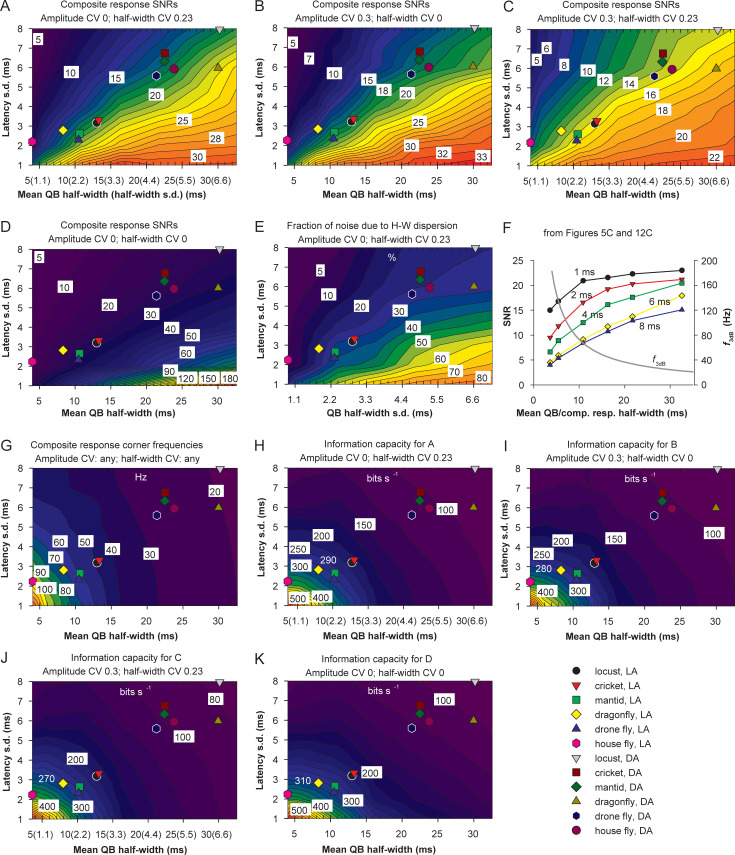
Interaction of noise sources and information capacity. (A-D) SNR as a function of variable mean QB half-width and latency dispersion in the presence of either half-width variability (A), or amplitude variability (B), or both the half-width and amplitude variabilities (C), or in the absence of any additional variability (D). Plots in A and C are based on 25 data points, and plots in B and D on 64 data points. (E) The relative contribution of noise due to variability in QB half-width in the absence of amplitude variability; the values were obtained by factoring out the noise in the absence of half-width dispersion (D) from the noise in the presence thereof (A). (F) Examples from Fig 12C of non-linear dependencies of SNR on the mean QB half-width at different latency standard deviations as indicated (i.e. horizontal sections of the plot), and the dependence of the corner frequency on the half-width of composite responses from Fig 5C; dual labeling on *x*-axis refers to mean QB half-width for the SNR functions and to the composite response half-width for the corner frequency function. (G) Corner frequency as a function of mean QB half-width and latency dispersion. (H-K) Information capacities for data in A-D, respectively, were obtained by multiplying the common corner frequency values from G by the logarithmically transformed values from A-D (see Eq 2); to illustrate the changes, numbers in white denote approximated information rates associated with light-adapted dragonfly photoreceptors.

There were four aspects to the results. Firstly, the noise levels decreased as the number of QBs in the composite response increased. Secondly, there were non-trivial differences between the overall relative noise levels of dark- and light-adapted states. In the locust, the overall noise decreased by about one-third at all summation levels after light adaptation (Fig 11B). In contrast, in the house fly the transduction noise increased (Fig 10F). As we show in the next section (Fig 12), the increase in the transduction noise level in the house fly can be explained by a more than 6-fold decrease in the mean QB half-width but only about 3-fold decrease in latency dispersion, whereas in the locust latency dispersion decreased to a greater extent than the mean QB half-width (also see Discussion). Thirdly, in the house fly, in both adaptation states, the transduction noise significantly exceeded the fraction of noise that was added when photon noise was introduced, especially in the light-adapted state (Fig 10F), whereas in the locust the differences were more ambiguous (Fig 11B). Fourthly, in the light-adapted house fly simulation, the change in the amplitude CV from 0.13 to 0.30 had very small effect on the transduction noise (Fig 10F), whereas a substantial increase can be seen in the locust (Fig 11B). These differences can be explained by non-linear interactions between the noise sources as discussed below (Fig 12).

Fig 10G summarizes the SNRs and Fig 10H the corner frequencies. Because corner frequency depends only on mean QB duration and latency dispersion, only one value for each adaptation state is shown in Fig 10H. Although the SNRs of the light-adapted house fly photoreceptors were relatively low (Fig 10G), the associated very high corner frequency of >140 Hz (Fig 10H) ensures that its expected information capacity if estimated using the Shannon–Hartley theorem (see below) would be much higher than that of both the dark-adapted house fly and the light-adapted locust photoreceptors (Fig 11C and 11D).

It should also be noted that situations when a fully dark-adapted photoreceptor is stimulated with a bright stimulus eliciting impulse responses consisting of ≥100 QBs or a fully light-adapted photoreceptor is stimulated with a dim-light stimulus evoking a ~5 QBs response are not typical. Thus, to properly compare “typical” noise levels associated with these adaptation states, it might be more pertinent to contrast the 100 QB summation level for the light-adapted photoreceptors with the 5 QB summation levels for the dark-adapted photoreceptors (Figs 10F and 11B).

### Interactions between the sources of transduction noise and information capacity

#### Interactions between the sources of noise

It followed from the analysis of transduction noise in three species (Figs 8F, 10F and 11B) that either some sources of noise, e.g. latency dispersion, dominate over others, or that different sources produce non-additive noise, as was suggested in previous studies [18,26]. We therefore performed a systematic analysis of interactions between the mean QB half-width and the three variabilities by constructing composite responses using various combinations of QB durations and dispersions. The general parameter space of such simulations is very extensive because any imaginable combination of the parameters can be implemented. Therefore, we limited our modeling by, firstly, using only the pairs of mean QB half-width/half-width standard deviation situated on the green regression line in Fig 9D, which fits the experimental data. All such QB sets were characterized by a CV of 0.23. Secondly, we used only two amplitude CVs, 0 and 0.3.

Several QB sets covering the range of half-widths from below 4 to over 32 ms were convolved with five latency distributions shown in Fig 5H, giving 25 unique data sets (Fig 12A–12D; in Fig 12B and 12H 64 data sets based on 8 QB sets and 8 latency distributions were used). The contour plots in Fig 12A–12D describe dependencies of the composite response SNR at the 100 QB summation level: (1) on the latency and half-width dispersions in the absence of variation in QB amplitude, by using bumps with varying shapes but same amplitudes (Fig 12A); (2) on the latency and amplitude dispersions in the absence of half-width variability, by using set-average QBs (Fig 12B); (3) on all three dispersions (Fig 12C); and (4) on the latency dispersion alone (Fig 12D). Mean QB half-widths and latency standard deviations estimated for the experimental data were added to these contour plots to visualize the “natural” range of parameter changes.

Because the signal in the SNR equaled the area under the average composite response and because in these stimulations the QB areas were kept the same, the SNR values are simply reciprocals of the noise estimates. The most important conclusion stemming from these figures (Fig 12A–12D) is that the mean QB duration is a SNR-boosting factor that offsets the noise produced by QB variabilities. It can be seen that in the presence of all three QB variabilities the noise-suppressing effect of QB duration was non-linear (Fig 12C and 12F).

When we introduced variabilities in half-width or amplitude in addition to the latency dispersion, the effects were surprisingly similar (Fig 12A, 12B and 12D). The SNR-suppressing effect of amplitude dispersion at CV 0.3 (Fig 12B) was slightly smaller than the effect of half-width dispersion at CV 0.23 (Fig 12A), suggesting that *per unit of CV the amplitude noise is less significant than the normal variability in QB duration*. Notice that for the DA locust photoreceptor (grey upside-down triangle) the SNR was ~14.8 (Fig 12C), i.e. higher than SNR 10.0 at amplitude CV 0.48 (Fig 11C). Importantly, because there was a broad variability both in QB half-width and amplitude dispersion measures for any fixed mean value (Fig 3D and 3F) the actual relative contributions of these two variabilities can vary from cell to cell. Moreover, for a given QB half-width, photoreceptors of *P*. *terraenovae* were generally characterized by a larger half-width dispersion than photoreceptors of *P*. *americana* (Fig 3D), indicating that the half-width dispersion is a more significant source of transduction noise in the fly than in the cockroach.

When a third dispersion was added to the data displayed in Fig 12A or 12B, the SNR was suppressed to dissimilar extents in different regions of the plot: by about one-third in the region of wide QBs and small latency dispersions (lower right corner of the plot) vs. virtually no change in the region of narrow QBs and large latency dispersions (upper left corner, Fig 12C). This implies that high latency dispersion subdues noise from two other QB sources.

Next, we determined the relative contributions of the latency and half-width dispersions to the total noise by comparing the noise levels between Fig 12A and 12D. Fig 12E shows that noise caused by variability in QB duration dominated, as expected, in the region of large half-width and small latency dispersions. Interestingly, the experimental data were clustered in the relatively narrow diagonal region of the plot, where the latency-derived noise was clearly predominant.

#### Corner frequency and information capacity

So far, we have shown that increased QB half-width, on the one hand, improves SNR, but, on the other hand, decreases corner frequency. As Fig 12F shows, these effects are non-linear and partly mutually exclusive. It was therefore important to evaluate how these two non-linearities shape the information capacity. We used a straightforward approach based on the Shannon-Hartley theorem with an assumption of linearity of the input and linearity and stationarity of the signal-transferring channel. Then the information capacity (*IC*) is simply:

$$IC=B\;\log_{2}\left(SNR+1\right)$$ (2)

where *B* is corner frequency. We obtained corner frequencies associated with the composite responses for values presented in Fig 12A–12D and plotted them as functions of mean QB half-width and latency dispersion in Fig 12G. The plot shows that high corner frequencies are sharply concentrated near the origin where both mean QB half-widths and latency dispersions are small.

By multiplying corner frequency values from Fig 12G by log_2_(*SNR*+1) values we obtained four sets of information capacity estimates (Fig 12H–12K). It can be seen that because of the logarithmic transformation, the information capacity is mainly determined by the corner frequency. As an example, the approximated information capacities of the light-adapted dragonfly photoreceptors (yellow diamond) are shown in the figure. All changes were in a 15% range, from about 270 bits s^-1^ when all three QB variabilities were present to about 310 bits s^-1^ when noise was solely due to latency dispersion.

## Discussion

In this study we first investigated how the phototransduction cascade in each microvillus shapes the QB properties and dispersions thereof, then studied the effects of QB duration and latency dispersion on the composite responses and their associated frequency-response functions, and finally explored how variabilities in QB latency, amplitude and duration produce noise and influence signal transfer.

### Insights from the parametric analysis

Using a model of a phototransduction cascade, we investigated relations between three main stages of the cascade and the properties of resulting QBs. A positive correlation between QB latency and amplitude can be expected in an amplifying cascade because more time is needed for greater amplification to take place. We found that while the times to peak for Gα, Gα-PLC and DAG indeed correlated positively with QB latencies (Fig 1F), and the *N*_max_ values for Gα, Gα-PLC and DAG correlated with QB amplitudes (Fig 1C), but not *vice versa* (Fig 1E and 1D), such correlations decreased progressively as the number of cascade stages between the correlated parameters increased. This was apparently due to the gradual “accumulation of stochasticity”, which, as a source of noise, destroys information describing deterministic relations between upstream and downstream parameters. Consequently, the correlations between the *N*_max_ and the corresponding times to *N*_max_ decreased similarly, from a fairly high correlation for Gα to the insignificant one for DAG (Fig 1H). Because ion channels open still downstream to DAG and also stochastically, this would further diminish any remaining causative connection. Plausibly, if the cascade was shorter by one or two stages, then a positive correlation between QB amplitude and latency could be observed.

However, as we demonstrated at the next stage of the analysis by *varying the average properties of the cascade*, a correlation that emerged between the median QB latencies and mean amplitudes for the different cascades in the model and different cells in the experiments was actually the opposite to that expected for an amplifying cascade, as greater amplitudes were associated with shorter latencies and *vice versa* (Fig 4C and 4D). We suggested that the relatively high QB amplitudes and small half-widths associated with faster cascades both in the model and experiments were due to more synchronous opening of more channels as a result of faster DAG accumulation (Fig 4M and 4N). This was supported by positive correlations between the latencies and half-widths (Fig 4A and 4B) and negative correlations between: half-widths and amplitudes in the model and *P*. *terraenovae* (Fig 4E and 4F), latencies and rise slopes (Fig 4G and 4H), and half-widths and rise slopes (Fig 4I and 4J).

### QB properties and signaling bandwidth

In our previous studies [28,32] we discovered a large variability in mean QB latencies and their dispersions in the normal photoreceptors of *P*. *americana*. Here we confirmed the findings for *P*. *terraenovae* and then investigated how median latency and its dispersion influence signal transfer (Fig 5). A consequence of the linear dependence of latency dispersion on median latency was inferior processing of higher frequencies by relatively slow photoreceptors due to the increased integration time of composite responses (see theoretical considerations in [5]). Therefore, if similar dependencies exist in other species, then, for instance, fast flyers would need to evolve mechanisms that lower both median latency and its dispersion. Indeed, our data on the variability in the fast fly *P*. *terraenovae* (Fig 3B) support this hypothesis. Its group-average median latency was 20.4 ± 5.3 ms (average of median latencies ± average latency scatter), substantially smaller than the *P*. *americana’s* 29.6 ± 6.6 ms. Although the differences in mean QB latency scatter values between the dark-adapted fly and the cockroach photoreceptors appear to be small, the fly phototransduction can speed up dramatically during light adaptation (Fig 6), with a decrease in all variabilities (Fig 10).

Using the model, we investigated how changes in activation parameters affected the median latency and its dispersion. We found that the more upstream was a parameter, the stronger was the effect of its change on latency dispersion (Fig 3A). Therefore, speeding up the cascade by modifying its activation upstream could be an evolutionary strategy to lower the latency dispersion. However, the slopes of the trend lines fitting the experimental values (Fig 3B) were more similar to the slopes of lines fitting values obtained by modifying two downstream activation parameters. Of these, the basal PLC activity cannot be increased but only suppressed resulting in longer latencies, whereas the TRP sensitivity to the gating factor(s) can probably be only sped up via calcium-dependent mechanisms resulting in faster QBs [31]. Thus elevation of intra-microvillar [Ca^2+^] during light adaptation, or physiological variability in [Ca^2+^] between different photoreceptors, or even some artificial damage-related variability during recordings, could contribute to the observed experimental variation.

Our finding that faster cascades, in addition to smaller latency dispersion, tend to generate slightly narrower QBs than slower cascades (Fig 4A and 4B) might have consequences for signal transfer (Fig 5C). Previous studies of macroscopic responses showed that QB duration decreases during light adaptation in *D*. *melanogaster* [9], and is generally shorter in the photoreceptors of species characterized by faster phototransduction and *vice versa* [4]. However, the differences were attributed to the accelerated QB termination. In contrast, here we demonstrated directly that faster cascades (or photoreceptors in experiments) produce QBs with faster onsets and intrinsically smaller half-widths than slower cascades (Fig 4). However, the decrease in mean QB half-widths associated with acceleration of phototransduction can account only for a small fraction of the QB narrowing observed during light adaptation (Fig 6A). The rest of the QB narrowing is likely due to acceleration of QB decay kinetics, which requires a global increase of a modulating factor, probably calcium [4]. For example, the QB termination kinetics could be accelerated in the model to the level observed in the light-adapted impulse responses of the house fly by a hundred-fold increase in PKC activity, which is Ca^2+^-dependent.

### A method to extract QB statistics from impulse responses

We exploited the intrinsic correlations between the QB properties discovered during data analysis and modeling by developing an algorithm to predict the main statistical properties of QBs from the properties of arbitrary multiphoton impulse responses to instantaneous stimuli (Fig 9).

The stages of the algorithm are: 1) prediction of median QB latency from the latency of the impulse response using the data from the parametric analysis; 2) prediction of latency scatter using the trend from the experimental data and the estimated median QB latency; 3) extraction of the mean QB half-width from impulse responses by using a non-linear dependence between the half-widths of the impulse response and QB latency standard deviation; 4) prediction of the half-width dispersion using the estimated mean QB half-width and the trend from the experimental data; and 5) prediction of the amplitude dispersion using the derived median latency and the modeled amplitude data.

There were two problems with the method, one concerning the dependence of latency scatter on median latency and another the amplitude CV. Because experimental data did not cover the interval from 0 to ~11 ms on the *x*-axis of Fig 3B, it was not clear if the latency dispersion was not eliminated before the median latency reached zero, i.e. if the phototransduction cascade at some point did not become deterministic. However, there are several arguments against such scenario. First, the correlation based on ~180 cells was very strong and regressed precisely onto the origin. Second, the simulations involving multiple activation parameter modifications produced median latencies of ~10 ms characterized by latency scatters consistent with the experimental trendline (Fig 9B). Third, the estimated group-average median latency of light-adapted house fly photoreceptors was 9.3 ms, not much smaller than the shortest median QB latency of *P*. *terraenovae* photoreceptors that equaled 11.8 ms. It is not likely that latency dispersion would collapse precipitously immediately outside of the experimentally determined latency range. Fourth, to eliminate or reduce the latency dispersion, the phototransduction process must cease being stochastic, that is, both the number of involved molecules and the reaction volume must increase drastically, which is clearly inconsistent with the situation in the microvillus. One could even argue that as the phototransduction cascade speeds up, it becomes more stochastic because presumably fewer Gα, Gα-PLC and DAG molecules are generated prior to channel opening.

The second problem was related to determining the QB amplitude CV in the light-adapted photoreceptors. Although we found a strong positive correlation in the model, the experimental data were only partly consistent (Table 1). Therefore, in our analysis of transduction noise in the light-adapted house fly and locust photoreceptors, in addition to the amplitude CVs, estimated from the relation established in the model (Figs 5J, 5K and 9C), we used CV of 0.3, the mean amplitude CV from *P*. *terraenovae*. It can be seen from Figs 10G and 11C that the differences in SNR were substantial, up to 40% in the case of the house fly photoreceptor. However, when the photon noise was added, the differences disappeared almost completely.

Despite these shortcomings, the proposed method allows reliable determination of median latencies, their scatter, and mean QB half-widths from small voltage impulse responses. This is particularly important in the study of elementary responses of photoreceptors in the fast-flying insect species characterized by very low membrane resistances, small membrane capacitances and small voltage QBs, which cannot be reliably isolated due to large surrounding membrane noise [14]. An experiment relying on our algorithm could involve stimulation of a dark-adapted photoreceptor with 1 ms or shorter pulses of light of decreasing intensity, until bump-like responses disappear in most of the trials. Averaging multiple recordings acquired at such intensity will reduce noise and reveal the hidden signal, a “pseudo-impulse response”, which then can be analyzed.

### QB properties and signaling accuracy in the model and in vivo

Several studies addressed the question of noise sources in the past. In the study of transduction and photon noises associated with impulse responses of dark-adapted locust photoreceptors [18], Laughlin and Lillywhite found that the relative contribution of photon noise decreased as the number of QBs in the impulse response increased and that the variabilities in QB latencies and amplitudes were major sources of the transduction noise. However, the noise due to variability in QB duration was not investigated.

In the study by Abshire and Andreou [27], the general conclusion on the quantitative dynamic relationships between the photon and transduction noise sources was consistent with both our and the Laughlin and Lillywhite study’s conclusions but QB variability was not explicitly researched.

In a recent modeling study, Parag and Vinnicombe concluded that mean phototransduction delay itself, and not the QB variabilities, was the most important source of noise [26], which appears to be inconsistent with our results. The study investigated introduction of noise at two signal processing stages: from a stimulus sequence to the actual absorbed photon sequence and then from the latter to the QB sequence. Essentially, this approach, although based on the minimum mean squared error distortion function, resembles that for determining information rate from coherence between input and output using a cross-correlation function [33]. The time delay term that emerges in both approaches as an information-destroying factor represents a well-known problem [34]. However, in the current study we focused exclusively on the deterioration of isolated impulse responses by the variabilities in the QB properties and numbers and not on the comparison of time series of inputs and outputs, and thus evaded the problem of time delay. Importantly, acceleration of phototransduction during light adaptation decreases the time delay and therefore would reduce its contribution to the distortion function. Such a decrease can be as large as four-fold, from over 40 ms for the dark-adapted *D*. *melanogaster* photoreceptors to ~10 ms for the light-adapted photoreceptors of house and drone fly species as we estimated in Fig 9A.

Here, our analysis of transduction noise demonstrated that all three variabilities are significant and non-additive sources of noise, and that their effect on SNR depends on the duration of the QB. The sources are non-additive because their recombination in our simulations did not result in proportional changes in the total level of noise (Fig 12A–12D).

As shown in Fig 12E, the contribution of half-width dispersion is clearly a function of the magnitude of the accompanying latency dispersion. Due to the linear dependence of latency dispersion on mean latency, 11 out of 12 experimental values from six species occupy a diagonal region in the plot, within which the relative contribution of half-width dispersion varies from 20 to 35% (Fig 12E). However, since these data represent voltage impulse responses, their estimated QB durations exceed those of the underlying current QBs, and this increases the relative role of half-width dispersion. Because membrane filtering increases QB duration but not the half-width CV, it can easily be envisioned that an increase in membrane resistance, e.g. caused by membrane hyperpolarization, could shift the distribution of the values in Fig 12E to the right and thus increase the noise-generating effect of the half-width dispersion. Therefore, the noise sources arising from the variabilities in QB latency and duration can interact dynamically and their relative contributions are situational.

Compared to two other QB variabilities, the unique feature of the latency dispersion was that it also decreases the corner frequency, by expanding the composite response. This effect was the stronger, the smaller was the ratio of mean QB half-width to a measure of latency dispersion (latency standard deviation or scatter) (Fig 5J). If during modeling of macroscopic responses, one would decrease the QB half-width while leaving the latency distribution intact [9], this will lead to underestimation of both the SNR and corner frequency, with possibly severe underestimation of information capacity. The complex interactions between QB duration and latency dispersion can be illustrated by the increase in the overall level of transduction noise after light adaptation in the house fly (Fig 10F) but not the locust (Fig 11B). In the house fly, the ratio of mean QB half-width to latency scatter decreased from 4.0 in the dark-adapted to 1.6 in the light-adapted photoreceptor. Despite the accompanying decrease in QB amplitude CV, the transduction noise increased (Fig 10F, center and right subpanels). In contrast, in the locust the ratio of mean QB half-width to latency scatter actually increased from 3.6 in the dark-adapted to 4.1 in the light-adapted photoreceptor. This, together with a decrease in the amplitude CV, caused a substantial decrease in the level of transduction noise (Fig 11B).

The contribution of the genuine QB amplitude noise also depends on the presence of other variabilities, including the photon noise. In fact, the latter could be considered as a greatly magnified form of the QB amplitude noise. Importantly, while the overall noise decreased as a function of $1/\sqrt{n}$, SNR even at the high summation level of 100 QBs did not exceed 10 when all sources of transduction noise and the photon noise were present (Figs 10G and 11C). This is consistent with the data from *P*. *americana* [28] and blow fly [33]. In order to improve SNR further by 10-fold, the number of QBs in the impulse response should increase by 100-fold, to the very high level of 10,000. If the stimulus lasts 1 ms, this rate would correspond to 10^7^ QBs per second. This is an extremely high response rate, and no photoreceptor can probably maintain it for over a fraction of second. Therefore, it cannot be expected that SNR can be further improved by simply increasing the number of QBs in the impulse response in still brighter light.

Arguably, the roles of the two noise sources, transduction and photon, in downstream signal processing have to depend on both the illumination level and the extent of signal summation. In the dim light both the transduction and photon noises will manifest in the variable shapes and sizes of individual impulse responses or QB averages in LMCs. It is unlikely that such variability could matter for the accuracy of vision until the responses to isolated pulses of light begin merging into a continuous graded voltage response. This, however, happens at different effective light intensities in diurnal and nocturnal species. For instance, integration of photoreceptor signals in diurnal flies appears to be limited by superposition of signals from six photoreceptors in neighboring facets, with the threshold of behavioral optomotor responses observed at ~1.7 QBs s^-1^ at the photoreceptor level [35]. In contrast, in the cockroach, the optomotor responses were observed at light levels corresponding to QB rates of ~0.1 QBs s^-1^ per photoreceptor [36], indicative of a much more extensive signal pooling than in the fly. As soon as the responses merge, the noise begins affecting the accuracy of visual perception. For instance, lowering the background light level markedly slows the speed of flying and changes the pattern of aerial maneuvers [37].

It should be noted that much of our signaling accuracy analysis is based on the linear system and additive Gaussian noise assumptions, e.g. we inferred SNRs from averages and subtractive noise estimates. While this approach seems to be adequate for comparison of noise sources that introduce irregularities to composite responses, such approximations can affect the “noise floor” of the cascade when compared to non-linear and Poisson approximations [26].

## Conclusions

The main conclusions of our study are the following:

1. Main QB parameters, when compared between cascades in the model and between individual photoreceptors in the experiments, are correlated and inherently depend on the cascade speed.
2. The QB duration and variabilities in QB latency, duration and amplitude determine the signaling bandwidth and contribute to SNR. The corner frequency is a reciprocal function of the impulse response duration, which, in turn, is a non-linear function of mean QB half-width and latency dispersion. The noise emitted by the cascade is also a reciprocal non-linear function of mean QB half-width.
3. It followed that to radically improve information capacity, e.g. to improve vision during day time, the mean QB half-width must decrease, at the cost of increased noise. Decrease in QB duration produces a sharp increase in corner frequency, which is a direct multiplier in the Shannon-Hartley’s information capacity equation. The accompanying decrease in SNR results in a less than proportional effect because of the logarithmic transformation.
4. In contrast, to reliably detect weak light signals in dim light the QB duration increases, at the expense of bandwidth. This is consistent with our previous observations [14].
5. To offset the increase in noise associated with narrowing of the QB, and decrease the impulse response-expanding effect of latency dispersion, the cascade accelerates. This decreases both latency and half-width dispersions, and possibly also the amplitude variability.
6. Our findings thus suggest that the widespread tendency of diurnal fliers to have fast phototransduction appears to be a general adaptation for the stochasticity containment, SNR improvement and bandwidth expansion.

In general, our results emphasize the principal differences in phototransduction cascade plasticity and light adaptation between the rhabdomeric photoreceptors of invertebrates and ciliary photoreceptors of vertebrates. In the latter, current response onsets do not accelerate with increasing stimulation, i.e. the rising phases of the light-induced current coincide at all intensities [38]. This is because the light response is initiated by the closing of transduction channels upon a decrease in the cytosolic cGMP, which keeps the channels open when cooperatively bound. While the times to peak of the ciliary photoreceptor photoresponse decrease with light adaptation, this is exclusively due to the progressively faster reopening of the channels, i.e. faster response termination. In essence, the phototransduction cascade in the ciliary photoreceptors is as fast as it can be and cannot be sped up. In contrast, photoresponses of insect photoreceptors do accelerate truly [10], and, as we showed here, this leads to a global improvement of performance.

## Methods

### Electrophysiology

We used *P*. *americana* data from the intracellular recording and patch-clamp datasets obtained in our previous studies [28,29]. We also acquired in vivo data from northern blow fly *Protophormia terraenovae*. The flies were caught in Oulu and kept at 22–24°C under a 18 h light/6 h dark regime. Young, 1–11 days post-eclosion females were used in intracellular recordings.

Fly preparation was done as follows. The animal was fixed and immobilized with wax. The reference electrode (Ag/AgCl wire) was inserted through a small cut in the thorax. A small hole for the recording electrode was made in the dorsal part of the left eye and sealed with silicon grease to prevent dehydration. Aluminosilicate microelectrodes (Harvard Apparatus) were manufactured using a laser puller (P-2000; Sutter Instruments) and filled with 2 M potassium acetate solution; microelectrodes had resistances in the range of 120–170 MΩ. Microelectrodes were inserted into the retina using a micromanipulator (SMX-model, Sensapex Oy, Oulu, Finland). Amplifier SEC-05L (NPI, Germany) was used for recordings with a custom MATLAB (MathWorks, Natick, MA, USA) acquisition software. Electrode capacitance was compensated. All recordings were made from the broadband green-sensitive photoreceptors.

Photoreceptors were stimulated with 1 ms flashes of a green LED with a peak wavelength of 525 nm. The light source was aligned with the photoreceptor’s optical axis. In the range of light intensities used for stimulation, dependence of LED light output on the driving current was linear. Light intensity was attenuated using a series of neutral density (ND) filters (Kodak, New York, NY, USA). Experiments were performed at room temperature 22–24°C. All cells used for analysis had resting potentials of < -45 mV and responded robustly to light.

### Model

To simulate QBs, we used a model created for *D*. *melanogaster* by Nikolic et al. [31,39] and modified in our previous study [28]. The model was run in the stochastic mode. A brief overview of the model is given in S1 Text and all changes to procure the control *P*. *americana* QB with brief rationales are provided in S1 Table.

### Data and statistical analysis

QBs were evoked by 1 ms flashes of low intensity light applied at a rate of 1 or 2 Hz. Stimulus intensity was adjusted to evoke single bumps with a probability between 0.09 and 0.56. QBs were detected and analyzed using a custom MATLAB script (by Paulus Saari) based on a template-matching algorithm. Every software-detected QB was checked manually. Dubious QBs or voltage responses apparently containing more than one QB were excluded. Amplitude, half-width and latency were estimated from individual fits using template-matching algorithm. Latency was measured as the interval between the stimulus and the moment the QB reached 10% of its maximum value. To control for the accuracy of bump detections and probability estimates, we first averaged all traces where no QBs were detected. If any residual signal was present in the mean response, the data were excluded from the analysis.

Both in simulations and experiments, QB latency distributions were best described by a probability density function of the lognormal distribution, although as mean latency decreased, the shape of the distribution approached the normal distribution. We fitted the latency distributions with lognormal equations and determined the medians and measures of scatter. The scatter interval can be defined as:

$$scatter=\frac{\left[\mu \times e^{\sigma }-\mu /e^{\sigma }\right]}{2}$$

where *μ* is the median and *σ* the standard deviation of the variable's natural logarithm obtained from the distribution fitting. The scatter as presented above is analogous to the standard deviation of a normally-distributed variable in the sense that the [*μ*/*σ*, μ*σ] interval for the lognormal and [*μ*-*σ*, *μ*+*σ*] interval for the normal distribution both contain approximately 2/3 of the probability. In our experiments, the standard deviations computed for the lognormally-distributed latencies differed little from the scatter values (the differences were in most instances within 10%). Because of the negligible differences between the two dispersion measures and for the sake of convenience, we used standard deviations in the various composite response reconstruction experiments presented.

In contrast, amplitude distributions obtained in simulations were characterized by variable skewness. For instance, as the cascade slowed the shape of the amplitude distribution evolved from a pronounced positive to negative skew. This did not allow using a lognormal distribution to describe the amplitude statistics. In the experiments, we observed both clearly lognormal and normal amplitude distributions. However, the former were caused by a loss of smaller amplitudes during QB detection since reliable separation of voltage bumps from the membrane noise of similar amplitude was usually impossible. Therefore, here we used mean values and standard deviations to describe the QB amplitude statistics. Likewise, the QB half-width and 25–75% rise slope distributions were generally normal and thus described using means and standard deviations.

Spearman’s rank order correlation coefficient (SROCC, ρ) was used in analyses of correlations. The data presented in figures can be found in the supporting S1 Data file.

## Supporting information

S1 Data (XLSX)

S1 Table Changes to the original *D*. *melanogaster* QB model [1, 2] to obtain the normal *P*. *americana* QB. (DOCX)

S1 Text Main equations of the model. (DOCX)
