# Supplementary material for: Speed of phototransduction in the microvillus regulates the accuracy and bandwidth of the rhabdomeric photoreceptor
Source: PLoS Comput Biol. 2020 Nov 16;16(11):e1008427. doi: 10.1371/journal.pcbi.1008427 (PMC7704055; doi:10.1371/journal.pcbi.1008427)
Supplement: S1 Table — (DOCX) [file pcbi.1008427.s002.docx]

**S1 Text**

Table 1. Changes to the original *D. melanogaster* QB model [[1](#_ENREF_1), [2](#_ENREF_2)] to obtain the normal *P. americana* QB.

| Parameter | Definition | Value in *D. mel.* | Value in *P. am.* | Justification and the effect of changes |
| --- | --- | --- | --- | --- |
| *w*_Ca_ | Ca^2+^ permeability | 0.877 | 0.675 | Reflect the putative *P. americana* TRPL to TRP channel expression ratio of 90:10; increases QB |
| *w*_Mg_ | Mg^2+^ permeability | 0.101 | 0.010 |  |
| *w*_Na_ | Na^+^ permeability | 0.011 | 0.315 |  |
| *w*_K_ | K^+^ permeability | 0.011 | 0 |  |
| *P*_1_ | Permeability of an open TRP channel | 1.0 | 2.7 | Increases total permeability; accounts for high conductance of TRPL channels |
| *I*_calx,sat_ | Saturation current for the *Calx* pumps | 12 pA | 8 pA | Reduces interference with the genuine QB current |
| *τ*_DAGdelay_ | Time constant for DAG to activate TRP | 12 ms | 4 ms | Decreases mean latency |
| *τ*_P,dark_ | Time constant for the decay of Gα-PLC in dark | 100 ms | 40 ms | These two parameters were altered to prevent reopening of transduction channels by the persisting elevated DAG |
| *τ*_Ddark_ | Time constant for the decay of DAG in dark | 80 ms | 40 ms |  |
| *τ*_GDP_ | Time for GDP-GTP exchange | 5 ms | 3 ms | Decreases mean latency |
| *D*_G_ | Diffusion constant of Gq protein | 1.2 µm^2^ s^-1^ | 3 µm^2^ s^-1^ | Decreases mean latency |
| *D*_Gα_ | Diffusion constant of Gqα subunit | 1.5 µm^2^ s^-1^ | 4 µm^2^ s^-1^ | Decreases mean latency |

References

1. Nikolic K, Loizu J, Degenaar P, Toumazou C. A stochastic model of the single photon response in *Drosophila* photoreceptors. Integrative Biology. 2010;2(7-8):354-70. doi: 10.1039/c0ib00031k.

2. Nikolic K, Loizu J. Drosophila Photo-transduction Simulator. Journal of Open Research Software. 2013;1(1):e1. doi: <http://doi.org/10.5334/503b9b1a69665>.
