# Supplementary material for: Speed of phototransduction in the microvillus regulates the accuracy and bandwidth of the rhabdomeric photoreceptor
Source: PLoS Comput Biol. 2020 Nov 16;16(11):e1008427. doi: 10.1371/journal.pcbi.1008427 (PMC7704055; doi:10.1371/journal.pcbi.1008427)
Supplement: S1 Text — (DOCX) [file pcbi.1008427.s003.docx]

**S1 Text. Main equations of the model**

To simulate QBs, we used a model previously created for *D. melanogaster*. Although the main equations of the model are presented below, the reader is encouraged to peruse the original paper [[1](#_ENREF_1), [2](#_ENREF_2)].

After a rhodopsin into metarhodopsin (M) conversion event, the rate of generating activated Gq protein (Gα) is:

$\nu_{1}\left( t \right)=\frac{1}{\tau_{\mathrm{coll}}\left( t \right)+\tau_{\mathrm{GDP}}}$ , (1)

where $\tau_{\mathrm{GDP}}$ is a characteristic time of GDP to GTP exchange, and $\tau_{\mathrm{coll}}$ is metarhodopsin – Gq collision time constant:

$\tau_{\mathrm{coll}}\left( t \right)=\frac{S_{\mathrm{mv}}}{\alpha_{1}D_{G}\left[ G_{\mathrm{tot}}-G\alpha-G\alpha PLC \right]}$ , (2)

where *S*_mv_ is the microvillus membrane area, *α*_1_ the metarhodopsin – Gq protein collision factor, D_G_ the diffusion rate for the trimer Gq protein, G_tot_ the total number of Gq proteins, GαPLC the activated PLC. Metarhodopsin is considered essentially immobile.

The change in the number of Gα can be expressed as:

$\frac{dG\alpha}{dt}=\nu_{1}\left( t \right)\left[ M\left( t \right)*\frac{\partial}{\partial t}f_{act}\left( t, \tau_{1} \right) \right]- \nu_{2}\left( t \right)G\alpha\left( t \right),$ (3)

where metarhodopsin availability function is convolved with the activation function $f_{act}\left( t,\tau\right)=1-e^{{-t}/\tau}$, describing the release of Gα after a delay. *ν*_2_ is the rate of GαPLC formation:

$\nu_{2}\left( t \right)=\alpha_{2}D_{G\alpha}\frac{{PLC}_{\mathrm{tot}}}{S_{\mathrm{mv}}\left[ 1+\sqrt{G\alpha PLC(t)/\pi} \right]^{2}}$ , (4)

where *α*_2_ the Gq – PLC is collision factor, $D_{G\alpha}$ the diffusion rate for Gα, and PLC_tot_ the total number of PLC molecules. The kinetics of GαPLC is given by:

$\frac{dG\alpha PLC}{dt}=\nu_{2}\left( t \right)G\alpha\left( t \right)-\frac{G\alpha PLC(t)}{\tau_{P}(\mathrm{Ca}^{2+})}$ (5)

The time constant $\tau_{P}$ describes decay of GαPLC. The decay is Ca^2+^-dependent but can also take place in the dark:

$\tau_{P}\left( \mathrm{Ca}^{2+} \right)=\tau_{P, dark}e^{-\beta_{2}A_{\mathrm{GAP}}\left( \mathrm{Ca}^{2+} \right)}$, (6)

where $\tau_{P, dark}$ is the basal GTPase activity at rest, $A_{\mathrm{GAP}}$ describes Ca^2+^-dependent GTPase activity, and *β*_2_ the activation constant for the action of GAP on PLC.

Activated PLC produces DAG at the rate:

$\nu_{3}\left( t,\mathrm{Ca}^{2+} \right)=\frac{1}{\tau_{coll,\mathrm{PIP}_{2}}\left( t \right)+\tau_{react,PLC}\left( \mathrm{Ca}^{2+} \right)}$ , (7)

where $\tau_{react,PLC}$ is a function of Ca^2+^-dependent PLC activity:

$\tau_{react,PLC}\left( \mathrm{Ca}^{2+} \right)=\tau_{\mathrm{pi}}e^{-\beta_{3}A_{\mathrm{PLC}}\left( \mathrm{Ca}^{2+} \right)}$ (8)

Here, *τ*_pi_ is the PLC activity at low concentrations of intracellular Ca^2+^, *β*_3_ the activation constant for PLC enzymatic activity and $A_{\mathrm{PLC}}$ is a function of free cytosolic Ca^2+^:

$A_{\mathrm{PLC}}\left( \mathrm{Ca}^{2+} \right)=\frac{\left[ \mathrm{Ca}^{2+} \right]_{\mathrm{free}}}{\left[ \mathrm{Ca}^{2+} \right]_{\mathrm{free}}+K_{\mathrm{pi}}}$ (9)

$\tau_{coll,\mathrm{PIP}_{2}}$ is a function describing Gα-PLC collisions with PIP_2_:

$\tau_{coll,\mathrm{PIP}_{2}}\left( t \right)=\frac{S_{\mathrm{mv}}}{\alpha_{3}D_{\mathrm{PIP}_{2}}\left[ \mathrm{PIP}_{2,\mathrm{tot}}-\mathrm{PIP}_{2,\mathrm{used}}(t) \right]}$, (10)

where *α*_3_ is the PIP_2_ – PLC collision factor, $D_{\mathrm{PIP}_{2}}$ the diffusion rate for PIP_2_, PIP_2,tot_ the total number of PIP_2_ and PIP_2,used_ the number of accumulated DAG molecules.

DAG lifetime is limited by Ca^2+^-dependent activity of DAG kinase (DGK):

$\tau_{\mathrm{DAG}}\left( \mathrm{Ca}^{2+} \right)=\tau_{D, dark}e^{-\beta_{4}A_{\mathrm{DGK}}\left( \mathrm{Ca}^{2+} \right)},$ (11)

where $\tau_{D, dark}$ is the time constant of DAG decay in the dark, *A*_DGK_ a function describing Ca^2+^-dependence of DGK, and *β*_4_ an activation constant for the action of DGK on DAG.

Accumulation of DAG triggers opening of TRP channels. The number of open channels was presented as the product between the number of channels in the active state *N*_act_ and the probability of a channel being in the open state:

$N_{open}\left( t \right)=N_{act}\left( t \right)\frac{{(1+K_{O}\left[ \mathrm{DAGd} \right])}^{n}}{{(1+K_{O}\left[ \mathrm{DAGd} \right])}^{n}+{(1+K_{C}\left[ \mathrm{DAGd} \right])}^{n}/Y_{0}\left( \mathrm{Ca}^{2+} \right)},$ (12)

where [DAGd] = [DAG(*t* - $\tau_{\mathrm{DAGdelay}}$)] is the concentration of delayed DAG molecules and $\tau_{\mathrm{DAGdelay}}$ is a delay constant for TRP channel activation,, *K*_O_ and *K*_C_ the ligand (DAG) binding affinity parameters for the open and closed channel protein conformations, respectively; *Y*_0_ the probability of channel opening in the dark; and *n* = 4 the number of channel subunits forming a functional channel. The model assumes that the basal activity of TRP channels is Ca^2+^- and calmodulin-dependent:

$Y_{0}\left( \mathrm{Ca}^{2+} \right)=Y_{0,\mathrm{dark}}+\left( Y_{0,\max}-Y_{0,\mathrm{dark}} \right) A_{\mathrm{cam}}\left( \mathrm{Ca}^{2+} \right)$ , (13)

where

$A_{\mathrm{cam}}\left( \mathrm{Ca}^{2+} \right)=\frac{\left[ \mathrm{Ca}^{2+} \right]_{\mathrm{tot}}}{\left[ \mathrm{Ca}^{2+} \right]_{\mathrm{tot}}+K_{\mathrm{camtrp}}}$ (14)

The current through the channels consists of currents of Ca^2+^, Na^+^, Mg^2+^ and K^+^ and can be described using the Goldman–Hodgkin–Katz equation:

$I_{\mathrm{TRP},q}\left( t \right)=N_{open}\left( t \right)w_{q}P_{1}z_{q}F\beta_{q}V_{m}\frac{{C_{q,\mathrm{in}}\left( t \right)-C}_{q,\mathrm{out}}\left( t \right)e^{-\beta_{q}V_{m}}}{1-e^{-\beta_{q}V_{m}}}$ (15)

where *P*_1_ is the permeability of an open channel, *N*_open_*w_q_P*_1_ = *P_q_* the permeability of open channels to ion species *q*, *z_q_* its valence and *V_m_* membrane potential. *β_q_* = *z_q_F*/*RT*, where *F* is Faraday constant, *R* gas constant and *T* temperature. *C_q,_*_in_ and *C_q,_*_out_ are the microvillar and outside concentrations of ion *q*, respectively. *P_q_*(*t*) can be obtained from the total permeability of microvillus membrane if permeability ratios for different ions *w_q_* are known.

The influx of calcium inactivates metarhodopsin, stopping generation of activated Gq proteins. As DAG is degraded by DGK, TRP channels close, aided by Ca^2+^-dependent activation of PKC:

$\nu_{PKC}\left( t \right)=\nu_{PKC, max}\left( \frac{\left[ \mathrm{DAG} \right]}{\left[ \mathrm{DAG} \right]+K_{PKC1}} \right)\left( \frac{\left[ \mathrm{Ca}^{2+} \right]_{free}}{\left[ \mathrm{Ca}^{2+} \right]_{free}+K_{PKC2}} \right)$, (16)

where $\nu_{PKC, max}$ is the maximum rate of TRP phosphorylation by PKC, $K_{PKC1}$ and $K_{PKC2}$ are the concentrations of DAG and Ca^2+^ for 50% activity of PKC.

The change in the number of active TRP channels *N*_act_ is:

$\frac{dN_{act}}{dt}={-\nu}_{PKC}\left( t \right){PKC}_{tot}N_{act}\left( t \right)+\nu_{dph}\left( \mathrm{Ca}^{2+} \right)(N_{TRP}-N_{act}\left( t \right)),$ (17)

where PKC_tot_ and $N_{TRP}$ are the total numbers of PKC molecules and TRP channels in the microvillus, *ν*_dph_ is the rate of dephosphorylation of TRP channels. Dephosphorylation rate of the channel is Ca^2+^- and calmodulin-dependent:

$\nu_{dph}\left( t \right)={\nu_{ph}e}^{-\beta_{5}A_{\mathrm{cam}}\left( \mathrm{Ca}^{2+} \right)}$ (18)

where *ν*_ph_ is the rate of TRP dephosphorylation in dark, *β*_5_ is deactivation constant for the relaxation of TRP and *A*_cam_ is Ca^2+^- induced activity of calmodulin.

References

1. Nikolic K, Loizu J, Degenaar P, Toumazou C. A stochastic model of the single photon response in *Drosophila* photoreceptors. Integrative Biology. 2010;2(7-8):354-70. doi: 10.1039/c0ib00031k.

2. Nikolic K, Loizu J. Drosophila Photo-transduction Simulator. Journal of Open Research Software. 2013;1(1):e1. doi: <http://doi.org/10.5334/503b9b1a69665>.
